# Etiologies, diagnostic work-up and outcomes of acute respiratory distress syndrome with no common risk factor: a prospective multicenter study

**DOI:** 10.1186/s13613-017-0281-6

**Published:** 2017-06-19

**Authors:** Nicolas de Prost, Tài Pham, Guillaume Carteaux, Armand Mekontso Dessap, Christian Brun-Buisson, Eddy Fan, Giacomo Bellani, John Laffey, Alain Mercat, Laurent Brochard, Bernard Maître, for the LUNG SAFE investigators, the ESICM trials group, the REVA network

**Affiliations:** 1 Service de Réanimation Médicale, DHU A-TVB, Hôpitaux Universitaires Henri Mondor, Assistance Publique-Hôpitaux de Paris, Créteil Cedex, 94010 France; 2 Groupe de Recherche Clinique CARMAS, Faculté de Médecine de Créteil, Université Paris Est Créteil, Créteil Cedex, 94010 France; 3 Groupe hospitalier des Hôpitaux Universitaires de l’Est Parisien, Pôle Thorax Voies aériennes, Unité de Réanimation médico-chirurgicale, Hôpital Tenon, AP-HP, Paris, France; 4 ECSTRA Team, Inserm, UMR 1153, Univ Paris Diderot, Sorbonne Paris Cité, Paris, France; 5 Inserm, UMR 915, Université Paris Est Créteil, Créteil Cedex, France; 6 Keenan Research Centre, Li Ka Shing Knowledge Institute, St Michael’s Hospital, Toronto, Canada; 7 Interdepartmental Division of Critical Care Medicine, University of Toronto, Toronto, Canada; 8 School of Medicine and Surgery, University of Milan-Bicocca, Monza, Italy; 9 Department of Emergency and Intensive Care, San Gerardo Hospital, Monza, Italy; 10 Departments of Anesthesia and Critical Care Medicine, Keenan Research Centre for Biomedical Science, St Michael’s Hospital, Toronto, Canada; 11 Departments of Anesthesia, Physiology and Interdepartmental Division of Critical Care Medicine, University of Toronto, Toronto, Canada; 12 Service de Réanimation Médicale, Centre Hospitalier Universitaire d’Angers, Angers, France; 13 Service de Réanimation Médicale, Hôpital Henri Mondor, 51, Avenue du Maréchal de Lattre de Tassigny, 94010 Créteil Cedex, France

**Keywords:** Respiratory distress syndrome, adult, Berlin definition, Outcomes, Diagnostic techniques and procedures

## Abstract

**Background:**

Patients meeting the Berlin definition for the acute respiratory distress syndrome (ARDS) might lack exposure to one or more “common” risk factors and exhibit different clinical phenotype and outcomes. We aimed to compare the clinical presentation and outcome of ARDS patients with or without risk factors, the impact on hospital mortality, and to assess the diagnostic work-up performed. The current study is an ancillary analysis of an international, multicenter, prospective cohort study (the Large Observational Study to Understand the Global Impact of Severe Acute Respiratory Failure, LUNG SAFE). Patients meeting ARDS criteria within 2 days of acute hypoxemic respiratory failure onset were included in the study and categorized as having risk factors or not. Outcomes were compared using propensity score matching.

**Results:**

Among 2813 patients, 234 (8.3% [7.3–9.3]) had no ARDS risk factor identified. These were older, had more frequent chronic diseases and presented with less severe SOFA and non-pulmonary SOFA scores (*p* < 0.001). Compared to other ARDS, CT scan (32.1 vs 23.9%, *p* < 0.001) and open lung biopsy (2.6 vs 0.2%, *p* < 0.001) were slightly more frequent but left heart filling pressures assessment was not (69.4 vs 68.4%, *p* > 0.99). Among ARDS with no risk factor, 45 patients (19.2%) had a specific diagnosis made. As compared to others, patients having ARDS with no risk factor had a lower ICU but not hospital mortality (34.6 vs 40.0%; *p* = 0.12). A matched cohort analysis confirmed the lack of significant difference in mortality.

**Conclusion:**

Eight percent of ARDS patients have no identified risk factor, 80% of whom have no etiological diagnosis made. The outcome of ARDS with no risk factor was comparable to other ARDS but few had a comprehensive diagnostic work-up, potentially leading to missed curable diseases.

*Trial registration* clinicaltrials.gov Identifier: NCT02010073

**Electronic supplementary material:**

The online version of this article (doi:10.1186/s13613-017-0281-6) contains supplementary material, which is available to authorized users.

## Background

The Berlin definition of the acute respiratory distress syndrome (ARDS) [1] was established to improve the reliability and the validity of ARDS diagnosis and to better classify patients according to the disease severity. This definition states that respiratory symptoms are to occur (or worsen) within seven days of exposure to one or more proposed “common” ARDS risk factor (e.g., pneumonia, gastric aspiration, extra-pulmonary sepsis, polytrauma). Because ARDS is a heterogeneous entity, previous studies have striven to individualize subgroups of patients based on the risk factors involved [2], the presence of diffuse alveolar damage or not on lung histological analysis [3, 4], the presence of direct or indirect lung injury [5] or, more recently, by defining subphenotypes using latent class analysis [6]. In this line, we have recently reported that 7.5% of ARDS patients had no common risk factors of the Berlin definition identified during ICU stay and that these patients showed different clinical features and outcomes than others [7], suggesting individualizing this subgroup of patients could be of interest. Of note, in this retrospective series, ARDS patients with no common risk factor identified had a comprehensive diagnostic work-up performed, including (but not limited to) chest CT scan and bronchoscopy with broncho-alveolar lavage (BAL) fluid examination in most cases, allowing for an etiological diagnosis to be obtained in 75% of cases, the main etiologies identified being immunologic, drug-induced, or neoplastic disorders.

To the best of our knowledge, no large-scale prospective study has assessed the clinical phenotype, the prevalence, the management and the outcomes of ARDS patients with no common risk factor of the Berlin definition. We therefore took advantage of the Large Observational Study to Understand the Global Impact of Severe Acute Respiratory Failure (LUNG SAFE) [8] and designed the ancillary ASTEROID study (ArdS with no Risk factor from the Berlin Definition), which was approved by the steering committee of the LUNG SAFE study before patient enrollment began. We aimed to (1) determine the prevalence of ARDS with no risk factor identified; (2) evaluate the diagnostic work-up performed and the etiologies of ARDS in this subgroup of patients; and (3) compare the clinical presentation and outcome of patients having ARDS with no risk factors to those of others.

## Methods

### Study design

The LUNG SAFE study was an international, multicenter, prospective cohort study [8]. As previously described [8], patients were enrolled during four consecutive winter weeks (February–March 2014 in the Northern hemisphere and June–August 2014 in the Southern hemisphere) within each participating intensive care unit (ICU, *n* = 459). Ethics committee approval was obtained by all participating ICUs and patient consent or ethics committee waiver of consent were required. ICUs were recruited by public announcements by the European Society of Intensive Care Medicine (ESICM), by national societies and networks endorsing the study, and by designated national coordinators (see Additional file 1: e-Appendix 1) [8]. The ASTEROID study is an ancillary study, which had been designed and approved by the steering committee of the LUNG SAFE study before the enrollment period began.

### Patients and data collection

All patients admitted to a participating ICU within the 4-week enrollment window and receiving invasive or non-invasive ventilation were included. Exclusion criteria were age <16 years or inability to obtain informed consent, when required. Following enrollment, patients were evaluated daily for acute hypoxemic respiratory failure, defined as the concurrent presence of (1) ratio of arterial oxygen tension to inspired fraction of oxygen (PaO_2_/F_I_O_2_) of 300 mmHg or less; (2) new pulmonary parenchymal abnormalities on chest x-ray or computed tomography; and (3) ventilatory support with continuous positive airway pressure, expiratory positive airway pressure, or positive end-expiratory pressure (PEEP) of 5 cmH_2_O or more. Day 1 was defined as the first day that acute hypoxemic respiratory failure criteria were met. Investigators were prompted to provide an expanded data set on the electronic case report form for days 1, 2, 3, 5, 7, 10, 14, 21, and 28 or at ICU discharge or death.

Patients meeting ARDS criteria on day 1 or 2 were included in the ASTEROID analysis and categorized as having “common” risk factors (Berlin definition [1]) or not depending on the identification of ARDS risk factors by managing physicians both upon ARDS diagnosis and during ICU stay (“late-identified risk factors”). Specific additional data were prospectively collected for patients having no risk factors (Additional file 1: e-Appendix 2). Patient outcomes included date of liberation from mechanical ventilation and vital status at ICU discharge and at either hospital discharge or at day 90, whichever occurred earlier. The primary end-point of the study was the prevalence of ARDS with no risk factor identified. Secondary endpoints were: (1) the comparison of clinical characteristics and outcomes of ARDS patients with and with no risk factor identified and (2) the diagnostic work-up performed and the etiologies of ARDS in this subgroup of ARDS with no risk factor identified.

### Statistical analysis

Continuous variables are reported as median (1st–3rd quartiles) or mean ± standard deviation (SD), as appropriate. Parametric or nonparametric tests were used according to the distribution of variables. Differences in categorical variables were analyzed using the Chi-square or the Fisher exact test or the McNemar test for matched patients, as appropriate. Continuous variables were compared using the Student’s *t* test or the Wilcoxon rank-sum test and the corresponding paired tests for matched patients, as appropriate. The relationship between the variable “no ARDS risk factor” and hospital mortality was assessed by the Kaplan–Meier method (patients were considered alive at day-60 if they were discharged alive before day-60) and by logistic regression. In-ICU and hospital mortality were compared between propensity-matched ARDS patients having one or more common risk factor identified or not. Covariates presumed to be associated with the presence of one or more ARDS risk factor or with hospital mortality were included in a multivariable analysis with “identification of ≥1 ARDS risk factor” as the dependent variable to determine the propensity score of this variable for each patient (see Additional file 1). Propensity score methods are usually used to reduce the bias in estimating treatments effects and the likelihood of confounding when analyzing non-randomized observational data [9]. In the current study, we used a propensity score method in order to assess the impact of the lack of ARDS risk factor on patient outcomes. Patients with one or more identified risk factor were thus matched with other patients according to the propensity score, using a 1:1 matching procedure. The relative change in the hazard of the ICU and hospital mortality were then assessed by regressing survival on the identification of a risk factor by using a univariate Cox proportional hazards model accounting for the matched nature of the sample. Two-tailed *p* values of less than 0.05 were considered statistically significant. Statistical analyses were performed with R3.2.3 (http://www.R-project.org).

## Results

### Prevalence of ARDS with no risk factor identified

**Fig. 1:**
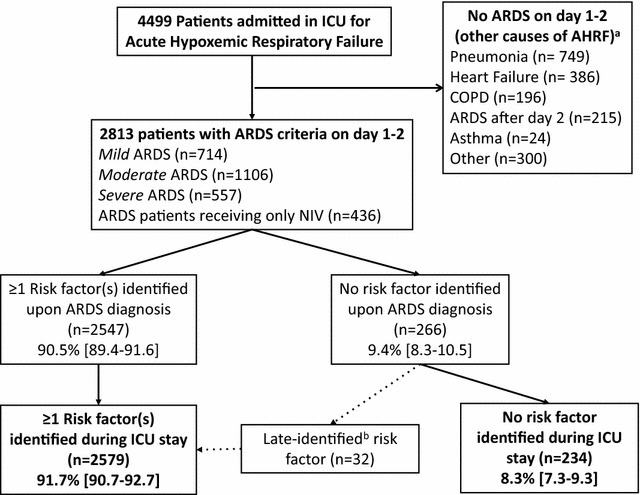
Flow of patients with acute hypoxic respiratory failure (AHRF) enrolled in the study. ICU, intensive care unit; ARDS, acute respiratory distress syndrome; COPD, chronic obstructive respiratory disease; ^a^ Patients could have more than one cause of acute hypoxic respiratory failure; ^b^ Factors identified during the course of ICU stay

A total of 4499 patients met the acute hypoxemic respiratory failure criteria of the LUNG SAFE study, of whom 2813 (62.5% [61.1–63.9]) had ARDS on day 1 or 2 (Fig. 1) and were included in the current analysis. In this cohort, 2547 patients (90.5% [89.4–91.6]) had a risk factor for ARDS identified upon ARDS diagnosis, while 266 (9.5% [8.4–10.6]) had no risk factor identified. Among these, 32 patients had a late-identified risk factor, leading to a prevalence of ARDS with no risk factor identified during ICU stay of 8.3% [7.3–9.3] (*n* = 234/2547).

### Clinical presentation of ARDS patients with and without risk factor

As compared with other patients with ARDS, those having no risk factor identified upon ARDS diagnosis were older (65 ± 15 vs 61 ± 17, *p* < 0.001) and had more comorbidities, including COPD (34.2 vs 20.4%, *p* < 0.001), diabetes (27.8 vs 21.2%, *p* = 0.025), cardiac (16.2 vs 9.8%, *p* = 0.003) or renal (16.2 vs 9.6%, *p* = 0.002) failure, but less frequent hematological malignancy (1.7 vs 5.2%, *p* = 0.027) (Table 1). In contrast, these patients presented with less severe pulmonary (ARDS severity) and non-pulmonary organ failures, as reflected by lower total (8.5 ± 3.7 vs 9.5 ± 4.1, *p* < 0.001) and non-pulmonary (5.3 ± 3.7 vs 6.3 ± 4.1, *p* < 0.001) SOFA scores. Differences in ventilator settings (PEEP, peak inspiratory pressure, F_i_O_2_ levels) likely reflected differences in ARDS severity between the two groups (Table 1). Except for a greater number of ICU bed per nurse in patients with no risk factors as compared to others (Additional file 1: e-Table 1), the characteristics of ICUs did not significantly differ between groups. The rate of ARDS with no risk factor ranged from 0 to 33% among countries (Additional file 1: e-Table 2).

**Table 1:** Baseline characteristics of patients with ARDS meeting ARDS criteria within 48 h of acute hypoxemic respiratory failure onset (*n* = 2813)

Parameters	ARDS patients with ≥1 risk factor identified (*n* = 2579)	ARDS patients with no risk factor identified (*n* = 234)	*p* value^a^
Age (years)	61 ± 17	65 ± 15	<0.01
Chronic disease			
COPD	527 (20.4)	80 (34.2)	<0.01
Diabetes	548 (21.2)	65 (27.8)	0.025
Immunoincompetence	544 (21.9)	40 (17.9)	0.17
Chronic cardiac failure	252 (9.8)	38 (16.2)	<0.01
Chronic renal failure	248 (9.6)	38 (16.2)	<0.01
Active neoplasm	210 (8.1)	22 (9.4)	0.58
Hematological malignancy	134 (5.2)	4 (1.7)	0.03
Chronic liver failure	106 (4.1)	6 (2.6)	0.33
Type of admission			<0.001
Medical	1979 (76.7)	179 (76.5)	
Postoperative	127 (4.9)	26 (11.1)	
Surgical	365 (14.1)	28 (12.0)	
Trauma	108 (4.2)	1 (0.4)	
ARDS severity			<0.001
Mild	651 (25.2)	63 (26.9)	
Moderate	1026 (39.8)	80 (34.2)	
Severe	524 (20.3)	33 (14.1)	
ARDS receiving only NIV	378 (14.7)	58 (24.8)	
Day 1 SOFA score^b^	9.5 ± 4.1	8.5 ± 3.7	<0.01
Day 1 non-pulmonary SOFA score^c^	6.3 ± 4.1	5.3 ± 3.7	<0.01
Worst SOFA score	11.2 ± 4.4	9.8 ± 4.1	<0.01
Worst non-pulmonary SOFA score	8.1 ± 4.2	6.8 ± 4.1	<0.01
FiO_2_	0.60 [0.45–0.80]	0.50 [0.40–0.80]	0.03
Total respiratory rate (1/min)	21.8 ± 8.9	21.3 ± 6.5	0.42
Tidal volume (mL/kg PBW)	7.7 ± 2.0	7.9 ± 2.3	0.20
In intubated patients	7.6 ± 1.9	7.7 ± 1.9	0.59
In NIV patients	8.3 ± 2.7	8.7 ± 3.2	0.52
Set PEEP (cmH_2_O)	8.2 ± 3.2	7.5 ± 2.4	<0.01
Peak pressure (cmH_2_O)	25.6 ± 8.8	23.5 ± 8.0	<0.01
Plateau pressure (cmH_2_O)^d^	23.2 ± 6.1	22.8 ± 5.4	0.65
Respiratory system compliance (mL/cmH_2_O)^d^	36.8 ± 22.1	33.5 ± 15.2	0.16
Driving pressure (cmH_2_O)^d^	14.7 ± 5.4	11.8 ± 6.0	0.67
Standardized minute ventilation (L/min)^e^	11.3 ± 5.4	11.9 ± 6.0	0.42
PaO_2_/FiO_2_ ratio (mmHg)	159 ± 67	169 ± 68	0.03
SpO_2_ (%)	96 [93−98]	96 [94–98]	0.18
PaCO_2_ (mmHg)	45.6 ± 15.3	48.8 ± 18.0	0.01
pH	7.33 ± 0.12	7.33 ± 0.12	0.90

Categorical variables are shown as n (%); Continuos variables are shown as median [1st-3rd quartiles] or mean ± standard deviation, as appropriate; ARDS: acute respiratory distress syndrome; COPD, chronic obstructive pulmonary disease

NIV, non-invasive ventilation; SOFA: Sequential Organ Failure Assessment; FiO_2_, inspired fraction of oxygen; PBW: Predicted Body Weight; PEEP: Positive End-Expiratory Pressure; SpO_2_: peripheral arterial oxygen saturation

^a^ *p* value represents comparisons across ARDS with or without known risk factor; Parametric or nonparametric tests were used according to the distribution of variables

^b^For all SOFA scores, where data points were missing, this value was omitted and the denominator adjusted accordingly

^c^For computing the non-pulmonary SOFA score, the pulmonary component of the score was omitted and the denominator adjusted accordingly

^d^Plateau pressure values are limited to patients in whom this value was reported (*n* = 732), and in whom either an assist control mode was used or, where a mode permitting spontaneous ventilation was used, the set and total respiratory rates were equal. Patients receiving high frequency oscillatory ventilation (HFOV) or extracorporeal membrane oxygenation (ECMO) were also excluded

^e^Standardized minute ventilation = minute ventilation * PaCO_2_/40 mmHg

### Diagnostic investigations and management of ARDS patients with and without risk factor

An objective assessment of left heart filling pressures was performed in 68.4% of patients having one or more ARDS risk factor identified, as compared to 69.4% of patients having no risk factor identified (*p* > 0.99) (Table 2), mainly using echocardiography. Still, even in the latter group, all investigators considered that acute hypoxemic respiratory failure was not fully explained by fluid overload. Among patients having no risk factor identified, a comparison of those who underwent an objective assessment of LHFP (*n* = 160), as required by the Berlin definition, or not (*n* = 74) revealed that the latter had less frequently known previous history of cardiac and chronic renal failure and more frequent immunoincompetence (Additional file 1: e-Table 3), suggesting they were less likely to have a mere cardiogenic pulmonary edema. Regarding diagnostic investigations (Table 2), patients having no risk factor identified underwent slightly more frequent chest CT scans (32.1% (*n* = 75) vs 23.9% (*n* = 617), *p* < 0.001) and open lung biopsy (2.6% (*n* = 6) vs 0.2% (*n* = 5), *p* < 0.001) than others. However, only a limited number of these patients underwent a more comprehensive diagnostic work-up, including bronchoscopy with BAL (*n* = 22, 9.4%), immunological tests (*n* = 12, 5.1%), and search for a pneumotoxic drug (*n* = 6, 2.6%).

**Table 2:** Management of ARDS patients meeting ARDS criteria within 48 h of acute hypoxemic respiratory failure onset (*n* = 2813)

Parameters	ARDS patients with ≥1 risk factor identified (*n* = 2579)	ARDS patients with no risk factor identified (*n* = 234)	*p* value^a^
*Diagnostic procedures*			
Assessment of LHFP	1780 (68.4)	160 (69.4)	>0.99
Echocardiography	1636 (63.4)	151 (64.5)	0.79
Pulmonary artery catheter	103 (4.0)	14 (6.0)	0.20
TTP	166 (6.4)	10 (4.3)	0.24
Other methods	155 (6.0)	12 (5.1)	0.69
Chest CT scan	617 (23.9)	75 (32.1)	<0.01
Number of chest CT scan	0 [0–0]	0 [0–1]	<0.01
Open lung biopsy	5 (0.2)	6 (2.6)	<0.01
*Treatment of ARDS*			
Neuromuscular blocking agents			
In the 1st 72 h of ARDS	440 (17.1)	26 (11.1)	0.02
At any time during ICU stay	517 (20.0)	33 (14.1)	0.04
Recruitment maneuvers			
In the 1st 72 h of ARDS	446 (17.3)	30 (12.8)	0.10
At any time during ICU stay	506 (19.6)	33 (14.1)	0.05
Prone positioning			
In the 1st 72 h of ARDS	134 (5.2)	8 (3.4)	0.30
At any time during ICU stay	190 (7.4)	11 (4.7)	0.17
ECMO			
In the 1st 72 h of ARDS	60 (2.3)	6 (2.6)	>0.99
At any time during ICU stay	70 (2.7)	8 (3.4)	0.67
Inhaled vasodilators			
In the 1st 72 h of ARDS	156 (6.0)	7 (3.0)	0.08
At any time during ICU stay	200 (7.9)	10 (3.8)	0.02
Corticosteroids			
In the 1st 72 h of ARDS	367 (14.2)	39 (16.7)	0.36
At any time during ICU stay	467 (18.1)	41 (17.5)	0.89

Categorical variables are shown as n (%)

ARDS, acute respiratory distress syndrome; CT, computed tomography; LHFP, left heart filling pressure; TTP, transpulmonary thermodilution device; ICU, intensive care unit; ECMO, extracorporeal membrane oxygenation

^a^ *p* value represents comparisons across ARDS with or without known risk factor; parametric or nonparametric tests were used according to the distribution of variables

Among the subgroup of ARDS with no risk factor, 45 (19.2%) patients eventually had an etiology for ARDS (Table 3), allowing for a targeted management, while 189 (80.8%) had no etiology identified during ICU stay. There was no significant difference in the number of autopsies performed in patients without as compared to with ARDS risk factors (9.4% (*n* = 6/64 decedents in ICU) vs 5.5% (*n* = 50/902), *p* = 0.26). Lung histological analyses obtained from open lung biopsy and autopsy revealed that diffuse alveolar damage (DAD), pulmonary edema, pneumonia, lung fibrosis and normal lung histology were the most common findings in both groups of patients (Table 4).

**Table 3:** Risk factors and etiologies identified for ARDS patients meeting ARDS criteria within 48 h of acute hypoxemic respiratory failure onset (*n* = 2813)

*Common risk factors* ^a^ *identified in 2579 ARDS patients* ^b^	
Pneumonia	1683 (65.2)
Non-pulmonary sepsis	455 (17.6)
Aspiration of gastric contents	402 (15.6)
Non-cardiogenic shock	214 (8.3)
Major trauma	112 (4.3)
Blood transfusion	111 (4.3)
Pulmonary contusion	87 (3.4)
Inhalational injury	70 (2.7)
Pancreatitis	59 (2.3)
Drug overdose	51 (2.0)
Pulmonary vasculitis^c^	38 (1.5)
Burn	8 (0.3)
Drowning	2 (0.1)
Others^d^	74 (2.9)
*Etiologies for ARDS in 234 patients having no common risk factor identified*	
Interstitial lung disease	9 (3.8)^e^
Drug-induced ARDS	8 (3.4)^f^
Massive hemoptysis	8 (3.4)
Pulmonary embolism	3 (3.4)
Diffuse alveolar hemorrhage	3 (3.4)
Alveolar proteinosis	3 (3.4)
Miscellaneous	11 (4.7)^g^
No etiology identified	189 (80.8)

Variables are shown as n (%)

ARDS: Acute Respiratory Distress Syndrome

^a^As listed in the Berlin definition of ARDS [1]

^b^Some patients had several risk factors

^c^ Including 24 patients who had both one or more common risk factors for ARDS and pulmonary vasculitis

^d^Includes conditions likely to be associated with common risk factors (e.g., major surgery (*n* = 23), coma/neurological disorders (*n* = 16), cardiogenic shock or cardiac surgery with cardiopulmonary bypass (*n* = 9), cardiac arrest (*n* = 8), and others (*n* = 18))

^e^With no specific diagnosis

^f^Including amiodarone (*n* = 4), bicalutamide (*n* = 1), warfarin (*n* = 1), chemotherapy agent (*n* = 1), not specified (*n* = 1)

^g^Including lung tumoral infiltration (*n* = 2), acute interstitial pneumonia (*n* = 1), Hemophagocytic Lymphohistiocytic syndrome (*n* = 1), lymphoproliferative disease with lung involvement (*n* = 1), graft versus host disease (*n* = 1), primary graft dysfunction (*n* = 1), gas embolism (*n* = 1), ovarian hyperstimulation syndrome (*n* = 1), Castleman’s disease (*n* = 1)

**Table 4:** Lung histological analysis results obtained from open lung biopsy or autopsy in patients with and without ARDS risk factor identified

Parameters	ARDS patients with ≥1 risk factor identified upon ARDS diagnosis (*n* = 2579)	ARDS patients with no risk factor identified upon ARDS diagnosis (*n* = 234)	*p* value
*Open lung biopsy*	5^a^ (0.2)	6 (2.6)	<0.001
DAD	0	2^b^	
Pulmonary fibrosis	0	2	
Normal lung histology	2	2	
*Autopsy* ^c^	50 (5.5)	6 (9.4)	0.26
Pulmonary edema	11	3	
Pneumonia	12	1	
DAD	10	2	
Pulmonary fibrosis	7	2	
Atelectasis	4	1	
Normal lung histology	2	3	
Intra-alveolar hemorrhage	1	1^d^	

DAD, diffuse alveolar damage; AIP, acute interstitial pneumonia

^a^Results were only available for 2 patients

^b^Including one patient with spumous macrophages related to cordarone-induced pneumonia

^c^Percentages have been computed with the number of decedents in the ICU as the denominator. Numbers in the column do not match with the total number of autopsies performed as some autopsy findings were unavailable and some patients had several histological findings

^d^Due to massive hemoptysis

Regarding ARDS management, the observed differences between the two groups reflected differences in ARDS severity, with patients from the no risk factor group receiving less neuromuscular blocking agents (14.1 vs 20.0%, *p* = 0.04) and inhaled vasodilators (3.8 vs 7.9%, *p* = 0.02). The rate of corticosteroids administration was not significantly different between groups (17.5 vs 18.1%, *p* = 0.89, Table 2).

### Patients with and without ARDS risk factor identified: prognostic comparison

Among survivors, patients with no risk factor for ARDS identified exhibited significantly lower durations of invasive mechanical ventilation as well as of ICU and hospital length of stay, as compared to ARDS survivors having at least one risk factor identified (Table 5).

**Table 5:** Outcome of ARDS patients meeting ARDS criteria within 48 h of acute hypoxemic respiratory failure onset (*n* = 2813)

Parameters	Before propensity score matching			After propensity score matching		
	ARDS patients with ≥1 risk factor identified (*n* = 2579)	ARDS patients with no risk factor identified (*n* = 234)	*p* value^a^	ARDS patients with ≥1 risk factor identified (*n* = 214)	ARDS patients with no risk factor identified (*n* = 214)	*p* value^a^
*Matched variables*						
Age (years)	61 ± 17	65 ± 15	<0.01	68 ± 14	66 ± 15	0.08
Immunoincompetence	544 (21.9)	40 (17.9)	0.17	34 (15.9)	38 (17.8)	0.67
Diabetes	548 (21.2)	65 (27.8)	0.025	62 (29.0)	61 (28.5)	0.99
COPD	527 (20.4)	80 (34.2)	<0.01	73 (34.1)	72 (33.6)	0.99
Chronic renal failure	248 (9.6)	38 (16.2)	<0.01	30 (14.0)	33 (15.4)	0.75
Chronic cardiac failure	252 (9.8)	38 (16.2)	<0.01	38 (17.8)	34 (15.9)	0.66
Chronic liver failure	106 (4.1)	6 (2.6)	0.33	5 (2.3)	6 (2.8)	0.99
pH	7.33 ± 0.12	7.33 ± 0.12	0.90	7.33 ± 0.12	7.33 ± 0.12	0.99
PaO_2_/FiO_2_ ratio (mmHg)	159 ± 67	169 ± 68	0.03	169 ± 65	168 ± 68	0.86
Set PEEP (cmH_2_O)	8.2 ± 3.2	7.5 ± 2.4	<0.01	7.7 ± 2.8	7.5 ± 2.4	0.43
Day 1 non-pulmonary SOFA score^c^	6.3 ± 4.1	5.3 ± 3.7	<0.01	5.6 ± 3.9	5.5 ± 3.7	0.70
NIV	378 (14.7)	58 (24.8)	<0.01	46 (21.5)	47 (22.0)	0.91
*Outcome variables*						
Invasive ventilation free days to day 28 (days)	9 [0–21]	14 [0–24]	<0.01	10 [0–21]	14 [0–24]	0.16
Duration of invasive ventilation (days)						
All patients	8 [4–15]	6 [3–14]	<0.01	8 [4–16]	6 [3–14]	<0.01
Surviving patients	9 [4–15]	5 [3–12]	<0.01	8 [4–16]	8 [4–14]	<0.01
ICU length of stay (days)						
All patients	10 [5–18]	8 [4–16]	<0.01	11 [5–20]	8 [4–16]	0.02
Surviving patients	11 [6–20]	8 [4–15]	<0.01	11 [5–22]	8 [4–16]	0.02
Decision of withholding or withdrawing treatments	639 (24.8)	63 (26.9)	0.52	53 (24.8)	59 (27.6)	0.56
ICU Mortality	902 (35.0)	64 (27.3)	0.02	67 (31.3)	59 (27.6)	0.44
Hospital length of stay (days)						
All patients	17 [8–32]	14 [8–26]	0.03	18 [10–32]	14 [8–26]	<0.01
Surviving patients	23 [13–40]	16 [11–31]	<0.01	25 [14–44]	16 [10–32]	<0.01
Hospital mortality	1029 (40.0)	81 (34.6)	0.12	81 (37.9)	75 (35.1)	0.60

Categorical variables are shown as n (%); Continuos variables are expressed as median [1st–3rd quartiles]

ARDS: Acute Respiratory Distress Syndrome; COPD, chronic obstructive pulmonary disease; NIV, non-invasive ventilation; SOFA: Sequential Organ Failure Assessment; FiO_2_, inspired fraction of oxygen; ICU, intensive care unit

^a^ *p* value represents comparisons across ARDS with or without known risk factor; Parametric or nonparametric tests were used according to the distribution of variables

**Fig. 2:**
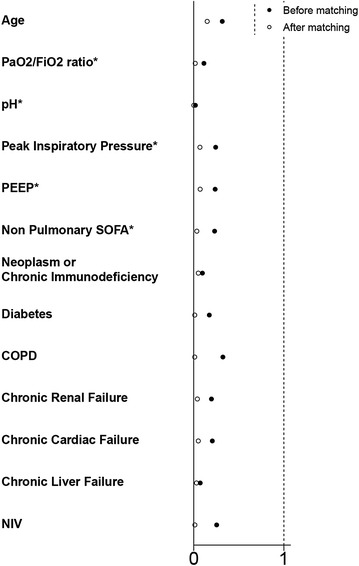
Covariates balances as measured by standardized differences across groups exposed or not to ARDS risk factors. Standardized differences are shown before (*closed circles*) and after (*open circles*) propensity score matching (including the variable “Peak inspiratory pressure”). PEEP, positive end-expiratory pressure; COPD, chronic obstructive pulmonary disease; NIV, non-invasive ventilation

**Fig. 3:**
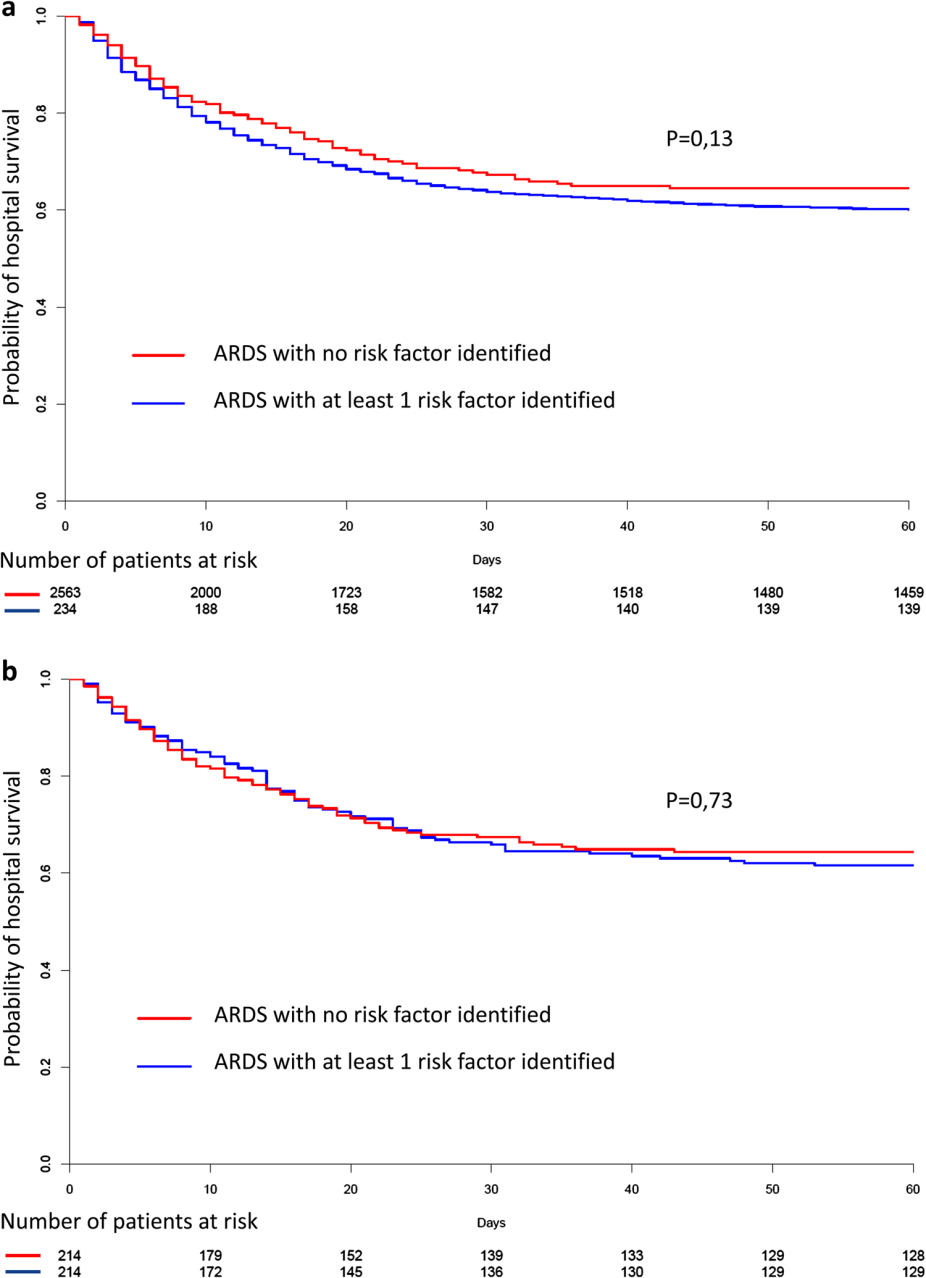
Kaplan–Meier curves of hospital survival probability in the whole (non-matched) cohort (**a**) and in the propensity score-matched cohort (**b**). ARDS patients with no risk factor identified (*red curve*) exhibited a non-significantly different probability of mortality during hospital stay, as compared with those having one or more risk factor identified (*blue curve*) both in the whole (**a**, *p* = 0.13, by the log-rank test) and the matched cohort (**b**, *p* = 0.73 by the Cox proportional hazard model)

The ICU mortality was significantly lower in ARDS patients with no risk factor than in others (27.3 vs 35.0%, *p* = 0.023), but in-hospital mortality was not significantly different (34.6 vs 40.0%, *p* = 0.12) (Table 5). Comparison of outcomes between propensity-matched ARDS patients having one or more common risk factor identified or not during ICU stay showed no significant difference (Table 5; Fig. 2; Additional file 1: e-Figure 1 for assessing the quality of propensity score matching), except for a shorter duration of hospital stay in the latter group as compared to the former one. Kaplan–Meier curves did not show significant differences in the probability of hospital death overtime in patients without vs. with identified risk factors both in the whole cohort (Fig. 3a, *p* = 0.13 by the log-rank test) and in the propensity score-matched cohort (Fig. 3b, *p* = 0.73 by the Cox proportional hazard model). By logistic regression analysis, there was also no significant relationship between the absence of ARDS risk factor and hospital mortality, both before and after propensity score matching (Table 6). Prognostic comparison of both groups was not altered by removing the variable “peak pressure” from the propensity score (Additional file 1: e-Figure 1, e-Table 4).

**Table 6:** Univariable logistic regression analysis assessing the relationship between the absence of identified ARDS risk factor and mortality before and after propensity score matching

	OR	95% IC	*p* value
ICU mortality *before* matching	0.70	0.52–0.94	0.02
ICU mortality *after* matching	0.84	0.55–1.27	0.40
Hospital mortality *before* matching	0.79	0.60–1.05	0.11
Hospital mortality *after* matching	0.89	0.60–1.31	0.55

ICU, intensive care unit

## Discussion

The main results of the current study are as follows: (1) in a large, prospective, international, multicenter cohort, eight percent of patients with ARDS had no risk factor identified at the end of their ICU stay; (2) about 80% of these had no etiological diagnosis made, and, despite slightly more chest CT scans and lung biopsy performed than in other patients, specific investigations aimed at diagnosing unusual causes of ARDS [10] were rarely performed. Assessment of left heart filling pressure was not more common than in other patients; and (3) although these patients presented a different clinical phenotype than others, with more comorbidities and less severe pulmonary and non-pulmonary organ failures, their outcomes were not significantly different from those of patients having one or more risk factor identified.

Eight percent of patients meeting ARDS criteria within 48 h of acute hypoxemic respiratory failure onset had no common risk factor for ARDS identified upon ARDS diagnosis or later during the stay. This figure is consistent with a previous retrospective study, which reported a prevalence of 7.5% [7], and confirms that this subgroup represents a significant proportion of patients with ARDS. Strikingly, an objective assessment of left heart filling pressures to rule out a pulmonary edema of the hydrostatic type was performed in less than 70% of cases, and no more frequently than in other patients, although this criterion is required by the Berlin definition of ARDS when no risk factor has been identified [1, 11]. Thus, one cannot exclude that some of these patients had in fact a pulmonary edema of the hydrostatic type, even if managing physicians declared that the acute respiratory failure was not entirely related to cardiac failure. Of note, the comparison of patients who underwent an objective assessment of LHFP to those who did not (Additional file 1: e-Table 3) revealed that the latter had less cardiac and renal comorbidities, suggesting they were at lower risk of developing a mere cardiogenic pulmonary edema, possibly explaining why physicians did not deem necessary to further explore cardiac function, even if required by the Berlin definition in this setting. Eighty percent of patients within this subgroup had eventually no risk factor nor etiology retrieved, demonstrating that in spite of an extensive literature on the unusual etiologies of ARDS (e.g., auto-immune and drug-induced diseases, organizing pneumonia, diffuse alveolar hemorrhage, lung tumor infiltration, acute pulmonary edema, pulmonary embolism) [7, 12–14] mainly coming from autopsy [4, 15] or lung biopsy studies [16–18], under “real-life” conditions, no etiological diagnosis is made in most of the cases. Indeed, in the current series, although chest CT scans were more frequently performed in patients with no risk factor than in others, key investigations aimed at identifying the cause of ARDS, including BAL cytological examination, immunological tests [10] and open lung biopsy [19] were performed in a minority of cases (9, 5, and 3%, respectively), despite the fact that investigators were prospectively requested to fill in a dedicated form addressing these aspects. This failure to identify the cause of ARDS in 80% of patients from the no risk factor group suggests that the individualization of patients’ management might have not been optimal. Indeed, specific interventions have been suggested to be beneficial in targeted cases. For instance, patients with auto-immune or drug-induced disorders, organizing pneumonia, or diffuse alveolar hemorrhage could benefit from anti-inflammatory treatments (e.g., corticosteroids) [7, 12, 13]. Interestingly, patients from the no risk factor group did not receive more frequent corticosteroids than others, both within 72 h of ARDS onset and during ICU stay, and the overall rate of corticosteroids administration was low (about 18%). In all, our findings suggest that the low diagnostic yield in the subgroup of ARDS patients with no risk factor precluded the administration of individualized treatments. Other differences observed in treatments administered (*i.e.*, neuromuscular blocking agents and inhaled vasodilators) and mechanical ventilation settings between both groups of patients likely resulted from differences in patients’ severity.

The current study provides a picture of the clinical presentation of ARDS patients with no risk factor identified and shows that these patients have a different clinical phenotype than others. Indeed, we not only observed differences regarding patients age and comorbidities, but also regarding the severity of ARDS and of associated organ failures, patients with no risk factor being less severe than others. Of note, such phenotype differences between patients without and with ARDS risk factors match those observed between patients with non-DAD vs. DAD ARDS, as recently shown in lung biopsy series [3, 16–18]. This observation corroborates the hypothesis that ARDS with no risk factor might be associated with a greater proportion of non-DAD lung histologies (sometimes termed “ARDS mimickers” [7, 13, 20]) than ARDS with one or more identified risk factors. However, we could not confirm this hypothesis due to the limited number of histological examinations performed in our study. In fact, the rate of open lung biopsy performed was strikingly low (*n* = 11/2813 in total, 0.4% of the whole cohort), illustrating that this procedure is exceptionally performed in ARDS patients. Yet, previous studies suggested that open lung biopsy, when performed in carefully selected patients, might not only allow for distinguishing patients with DAD and non-DAD ARDS, as discussed above, but have also been reported to allow for diagnoses to be made in 84% of cases and to alter management in 73% [21]. Thus, we believe that in patients with persisting ARDS, when a comprehensive diagnostic work-up has been performed, including—but not limited to—bronchoscopy with BAL fluid examination with extended microbiological and cytological analyses, CT scan examination, and immunological tests, and when no definite diagnosis has been retained, there might be room for open lung biopsy [12]. Of note, studies aiming at assessing the performances of a diagnostic work-up/algorithm in ARDS patients have, to the best of our knowledge, never been performed and would certainly be welcome.

In the current study, patients with no ARDS risk factor identified had a lower ICU mortality but no statistically different hospital mortality, as compared with others. A propensity score-matched analysis further confirmed the lack of significant differences both in ICU and hospital mortality between patients with and without ARDS risk factors, suggesting that the ICU mortality difference observed in the whole cohort was likely related to associated factors rather than to the lack of ARDS risk factor identification per se. Such results contrast with a previous study [7], in which we had reported a significant relationship between the lack of ARDS risk factor and mortality. Several factors might account for these conflicting results including differences in case mix and in patients’ management, particularly regarding the diagnostic work-up performed and the administration of corticosteroids in patients having no risk factor identified. We can only speculate on whether a more extensive work-up could have provided more diagnosis responding to specific therapies [10, 22, 23]. Indeed, the lack of specific procedures in most patients of the no risk factor group (e.g., BAL, lung biopsy) suggests that other diagnoses may have been missed. A special effort should be made in the future to target this group through guidelines and recommendations.

Our study has the following limitations: First, the current study is an ancillary study of a large prospective, international, multicenter study [8], and was thus not specifically designed for studying the subgroup of ARDS patients with no risk factor. However, the current study had been designed and approved before the enrollment period began and a dedicated section of the online case report form had been elaborated a priori for the prospective collection of data pertaining to ARDS with no risk factor; Second, although this study was performed in 459 ICUs worldwide, selection bias related to participating centers might have occurred, thereby limiting the generalizability of the findings; Third, we lacked access to the source data, implying that patients with ARDS might have been missed and that risk factors for ARDS might have been omitted, and thus some patients misclassified.

## Conclusion

 About eight percent of patients with ARDS have no risk factor identified and exhibit a different clinical phenotype, with more pre-existing chronic illnesses and less severe pulmonary and non-pulmonary organ failures than others. Thirty percent of these patients lacked an objective assessment of left heart filling pressure while 80% of them had no accurate etiological diagnosis made with most of them undergoing a limited diagnostic work-up, likely limiting the individualization of patient management. Compared to patients with ARDS of similar severity but with identified risk factors, their outcome was similar. Prospective studies should propose a specific work-up and test whether this can improve outcomes.

## Additional file

**Additional file 1.** Online supplement (Methods and Results), list of LUNG SAFE investigators (e-Appendix 1) and supplemental form to be filled for patients with no ARDS risk factor identified (e-Appendix 2).

## Abbreviations

ARDS: acute respiratory distress syndrome
BAL: broncho-alveolar lavage
ICU: intensive care unit
PEEP: positive end-expiratory pressure
