# Supplementary material for: Etiologies, diagnostic work-up and outcomes of acute respiratory distress syndrome with no common risk factor: a prospective multicenter study
Source: Ann Intensive Care. 2017 Jun 19;7:69. doi: 10.1186/s13613-017-0281-6 (PMC5476531; doi:10.1186/s13613-017-0281-6)
Supplement: Supplementary file 1 — Additional file 1. Online supplement (Methods and Results), list of LUNG SAFE investigators (e-Appendix 1) and supplemental form to be filled for patients with no ARDS risk factor identified (e-Appendix 2). [file 13613_2017_281_MOESM1_ESM.docx]

**Additional file 1**

**Etiologies, diagnostic work-up and outcomes of ARDS with no common risk factor**

**METHODS**

***Patients and data collection***

The variable “immunoincompetence” was defined as the presence of one of the following conditions: solid tumor which has not been resected or in remission,which is still requiring treatment or with metastasis, active hematologic neoplasm still requiring  treatment, or viral immunosuppression, neoplasic disease, immunosuppressive drugs (including steroids), chemotherapy or congenital immunosuppresion illness.

***Statistical analysis***

Propensity score: Covariates presumed to be associated with the presence of one or more ARDS risk factor or with hospital mortality were included in a multivariable analysis with “identification of ≥ 1 ARDS risk factor” as the dependent variable to determine the propensity score of this variable for each patient. A large number of variables were included into the multivariable model so that to increase its ability to effectively adjust for confounding (Haukoos, JAMA 2015). The independent variables selected were as follows: age, variables reflecting the severity of the acute respiratory failure (PaO_2_/FiO_2_ ratio and PEEP level on the first day of ARDS), variables reflecting the severity of non-pulmonary organ failures (non-pulmonary SOFA score on the first day of ARDS), pH (on the first day of ARDS), (on the first day of ARDS), , immune-incompetence (including active neoplasm and hematologic malignancy), diabetes, COPD, chronic renal failure, chronic cardiac failure, chronic liver failure, use of non-invasive (as opposed to invasive) mechanical ventilation. In order to test its impact on outcome, the variable “Peak Inspiratory Pressure (on the first day of ARDS)” was entered in the first propensity score. Respiratory system compliance could not be included in the model since this variable was only available in 732 patients. A second analysis was also performed without entering this variable into the propensity score. Standardized differences before and after matching were plotted (Figure 2 of the main manuscript and Figure 1 of the supplementary material).

Case-matching procedure: Patients with one or more identified risk factor were matched with other patients according to the propensity score, using a 1:1 matching procedure without replacement and a caliper width of 0.2 (*i.e.*, 0.2 x standard deviation of the logit of the propensity score, see online supplement for further details). The relative change in the hazard of the ICU and hospital mortality were then assessed by regressing survival on the identification of a risk factor by using a univariate Cox proportional hazards model accounting for the matched nature of the sample.

R libraries: Statistical analyses have been run using the following R libraries: “prettyR”, “psy”, “ResourceSelection”, “ROCR”, “cvAUC”, “survival”, “Matching”

e-Table 1. Characteristics of intensive care units (ICUs) according to the identification of ARDS risk factors or not.

|  | **ARDS patients with ≥1 risk factor identified**  **(n=2579)** | **ARDS patients with no risk factor identified**  **(n=234)** | **P value** |
| --- | --- | --- | --- |
| ICU beds | 17 [12-24] | 18 [12-26] | 0.05 |
| ICU bed per Physician | 4.7 [2.7-9.4] | 5.3 [2.8-9.5] | 0.23 |
| ICU bed per Nurse | 1.4 [1.0-2.0] | 1.7 [1.1-2.1] | <0.01 |

e-Table 2. Numbers and rate of ARDS patients with no risk factor identified among participating countries.

|  | **Number of ARDS with no risk factor identified** | **Total number of ARDS** | **Rate of ARDS with no risk factor identified** |
| --- | --- | --- | --- |
| Albania | 0 | 3 | 0% |
| Argentina | 5 | 77 | 6% |
| Australia | 12 | 125 | 9% |
| Austria | 1 | 3 | 25% |
| Belgium | 2 | 36 | 5% |
| Brazil | 2 | 18 | 10% |
| Brunei Darussalam | 1 | 5 | 17% |
| Canada | 13 | 88 | 13% |
| Chile | 1 | 37 | 3% |
| China | 6 | 189 | 3% |
| Colombia | 10 | 39 | 20% |
| Costa Rica | 0 | 13 | 0% |
| Czech Republic | 1 | 2 | 33% |
| Denmark | 1 | 22 | 4% |
| Ecuador | 0 | 1 | 0% |
| France | 24 | 347 | 6% |
| Germany | 0 | 17 | 0% |
| Greece | 0 | 18 | 0% |
| Guatemala | 0 | 2 | 0% |
| India | 2 | 56 | 3% |
| Iran | 0 | 9 | 0% |
| Ireland | 2 | 101 | 2% |
| Italy | 16 | 125 | 11% |
| Japan | 9 | 81 | 10% |
| Latvia | 0 | 5 | 0% |
| Lebanon | 0 | 1 | 0% |
| Malaysia | 0 | 0 | 0% |
| Mexico | 1 | 40 | 2% |
| Morocco | 1 | 12 | 8% |
| Netherlands | 9 | 59 | 13% |
| New Zealand | 0 | 27 | 0% |
| Norway | 4 | 33 | 11% |
| Peru | 0 | 5 | 0% |
| Philippines | 0 | 5 | 0% |
| Poland | 0 | 5 | 0% |
| Portugal | 7 | 80 | 8% |
| Romania | 3 | 19 | 14% |
| Russian | 0 | 3 | 0% |
| Saudi | 1 | 57 | 2% |
| Serbia | 0 | 5 | 0% |
| South Africa | 1 | 19 | 5% |
| Spain | 29 | 259 | 10% |
| Sweden | 8 | 65 | 11% |
| Switzerland | 2 | 22 | 8% |
| Tunisia | 2 | 15 | 12% |
| Turkey | 0 | 15 | 0% |
| United Kingdom | 17 | 322 | 5% |
| United States | 38 | 306 | 11% |
| Uruguay | 3 | 20 | 13% |

**e-Table 3. Baseline characteristics of patients with ARDS having no risk factor identified (n=234), whether they underwent an objective assessment of left heart filling pressures (LHFP, n=160) or not (n=74).**

| **Parameters** | **LHFP not objectively assessed**  **(n=74)** | **LHFP objectively assessed**  **(n=160)** | **P value^a^** |
| --- | --- | --- | --- |
| **Age**, **years** | 69 [58-76] | 68 [57-77] | 0.85 |
| **Chronic disease**  COPD  Diabetes  Immunoincompetence  Chronic cardiac failure  Chronic renal failure  Active neoplasm  Hematological malignancy  Chronic liver failure | 24 (32.4)  24 (32.4)  19 (25.7)  6 (8.1)  6 (8.1)  11 (14.9)  2 (2.7)  5 (6.8) | 56 (35.0)  41 (25.6)  21 (13.1)  32 (20.0)  32 (20.0)  11 (6.9)  2 (1.2)  1 (0.6) | 0.81  0.36  0.029  0.035  0.035  0.088  0.80  0.013 |
| **Type of admission**  Medical  Postoperative  Surgical  Trauma | 53 (71.6)  13 (17.6)  8 (10.8)  0 (0) | 128 (78.7)  13 (8.1)  20 (12.5)  1 (0.6) | 0.17 |
| **ARDS Severity**  Mild  Moderate  Severe  ARDS receiving only NIV | 19 (25.7)  25 (33.8)  9 (12.2)  21 (28.4) | 44 (27.5)  55 (34.4)  24 (15.0)  37 (23.1) | 0.82 |
| **Day 1 SOFA score^b^** | 8 [6-11] | 8 [6-11] | 0.95 |
| **Day 1 Non-pulmonary SOFA score^c^** | 5 [2-8] | 5 [2-7] | 0.89 |
| **Worst SOFA score** | 9 [7-12] | 10 [7-13] | 0.41 |
| **Worst non-pulmonary SOFA score** | 6 [4-9] | 7 [4-10] | 0.33 |
| **FiO_2_** | 0.50 [0.40-0.70] | 0.50 [0.40-0.80] | 0.80 |
| **Total respiratory rate, 1/min** | 20 [17-27] | 20 [16-25] | 0.11 |
| **Tidal volume, mL/kg PBW** | 7.7 [6.5-9.9] | 7.7 [6.6-8.5] | 0.21 |
| **Set PEEP, cmH_2_O** | 8 [5-10] | 8 [5-8] | 0.58 |
| **Peak pressure, cmH_2_O** | 22 [18-26] | 23 [18-28] | 0.27 |
| **Standardized minute ventilation, L/min^e^** | 11.1 [8.3-14.7] | 10.1 [7.7-13.8] | 0.18 |
| **PaO_2_/FiO_2_ ratio, mmHg** | 166 [111-215] | 169 [108-224] | 0.49 |
| **SpO_2_, %** | 96 [92-99] | 96 [94-98] | 0.34 |
| **PaCO_2_, mmHg** | 43.8 [36.6-54.9] | 42.5 [37.4-56.2] | 0.95 |
| **pH** | 7.35 [7.28-7.41] | 7.34 [7.26-7.42] | 0.27 |

Categorical variables are shown as n (%); Continous variables are shown as median [1^st^-3^rd^ quartiles] or mean±standard deviation, as appropriate; ARDS: Acute Respiratory Distress Syndrome; COPD, chronic obstructive pulmonary disease; NIV, non-invasive ventilation; SOFA: Sequential Organ Failure Assessment; FiO_2_, inspired fraction of oxygen; PBW: Predicted Body Weight; PEEP: Positive End-Expiratory Pressure; SpO_2_: peripheral arterial oxygen saturation; ^a^ P value represents comparisons across ARDS with or without known risk factor; ^b^ For all SOFA scores, where data points were missing, this value was omitted and the denominator adjusted accordingly; ^c^ For computing the non-pulmonary SOFA score, the pulmonary component of the score was omitted and the denominator adjusted accordingly;

**e-Table 4. Univariable logistic regression analysis assessing the relationship between the absence of identified ARDS risk factor and mortality before and after propensity score matching (Peak pressure removed from the model)**

|  | OR | 95% IC | P value |
| --- | --- | --- | --- |
| ICU mortality **before** matching | 0.70 | 0.52-0.94 | 0.02 |
| ICU mortality **after** matching | 0.68 | 0.46-1.01 | 0.06 |
| Hospital mortality **before** matching | 0.79 | 0.60-1.05 | 0.11 |
| Hospital mortality **after** matching | 0.69 | 0.47-1.01 | 0.06 |

ICU, intensive care unit

**e-Figure 1**

**Covariates balances as measured by standardized differences across groups exposed or not to ARDS risk factors, before (black circles) and after (red circles) propensity score matching.** The variable “Peak pressure” was removed from the propensity score. PF, PaO_2_/FiO_2_ ratio; PEEP, positive end-expiratory pressure; COPD, chronic obstructive pulmonary disease; NIV, non-invasive ventilation

**e-Appendix 1: List of LUNG SAFE investigators**

**List of LUNG SAFE Investigators**

**NATIONAL COORDINATORS:**

**Argentina:** Fernando Rios; **Australia/New Zealand**: Frank Van Haren; **Bangladesh**: Mohammad Omar Faruq; **Belgium:** Sottiaux T, Depuydt P; **Bolivia:** Fredy S Lora; **Brazil:** Luciano Cesar Azevedo; **Canada:** Eddy Fan; **Chile:** Guillermo Bugedo ; **China:** Haibo Qiu; **Colombia**: Marcos Gonzalez; **Costa Rica:** Juan Silesky; **Czech Republic:** Vladimir Cerny; **Denmark:** Jonas Nielsen; **Ecuador:** Manuel Jibaja; **France:** Tài Pham; **Germany:** Hermann Wrigge; **Greece:** Dimitrios Matamis; **Guatemala:** Jorge Luis Ranero; **Hong Kong:** Charles Gomersall; **India:** Pravin Amin; **Iran:** S.M. Hashemian; **Ireland:** Kevin Clarkson; **Italy:** Giacomo Bellani; **Japan:** Kiyoyasu Kurahashi; **Korea:** Younsuck Koh; **Mexico:** Asisclo Villagomez; **Morocco:** Amine Ali Zeggwagh; **Netherlands**: Leo M Heunks; **Norway**: Jon Henrik Laake ; **Pakistan:** Waqar Kashif ; **Panama:** Jorge Synclair; **Philippines:** Jose Emmanuel Palo ; **Portugal:** Antero do Vale Fernandes; **Romania:** Dorel Sandesc; **Saudi Arabia:** Yaasen Arabi; **Serbia:** Vesna Bumbasierevic; **Spain**: Nicolas Nin, Jose A Lorente; **Sweden**: Anders Larsson; **Switzerland**: Lise Piquilloud; **Thailand**: Boonsong Patjanasoontorn ; **Tunisia**: Fekri Abroug; **United Kingdom:** Daniel F McAuley, Lia McNamee; **Uruguay**: Javier Hurtado; **USA**: Ed Bajwa; **Venezuela:** Gabriel Démpaire;

**NATIONAL SOCIETIES/NETWORKS ENDORSING THE STUDY:**

ANZICS Clinical Trials Group, Réseau Européen de Recherche en Ventilation Artificielle (ReVA Network); Irish Critical Care Trials Group; Société de Réanimation de Langue Française (SRLF); Société Française d’Anesthésie et de Réanimation (SFAR); Società Italiana Anestesia, Analgesia, Rianimazione e Terapia Intensiva (SIAARTI); The Japanese Society of Intensive Care Medicine (JSICM); Nonprofit Organization Japanese Society of Education for Physicians and Trainees in Intensive Care (JSEPTIC); UK Intensive Care Society.

**STUDY COORDINATION:**

Guy M Francois (European Society of Intensive Care Medicine, Brussels, Belgium)

**DATA REVISION AND MANAGEMENT:**

Francesca Rabboni (University Of Milan-Bicocca, Monza, Italy), Fabiana Madotto (University Of Milan-Bicocca, Monza, Italy), Sara Conti (University Of Milan-Bicocca, Monza, Italy)

**SITE INVESTIGATORS BY COUNTRY:**

**ALBANIA:** Uhc Mother Theresa (Tirana): Hektor Sula, Lordian Nunci; University Hospital Shefqet Ndroqi (Tirana): Alma Cani;

**ARGENTINA:** Clinica De Especialidades (Villa Maria): Alan Zazu ; Hospital Dr Julio C. Perrando (Resistencia): Christian Dellera, Carolina S Insaurralde; Sanatorio Las Lomas (San Isidro, Buenos Aires): Risso V Alejandro; Sanatorio De La Trinidad San Isidro (San Isidro): Julio Daldin, Mauricio Vinzio; Hospital Español De Mendoza (Godoy Cruz - Mendoza): Ruben O Fernandez; Hospital Del Centenario (Rosario): Luis P Cardonnet, Lisandro R Bettini; San Antonio (Gualeguay (Entre Rios)): Mariano Carboni Bisso, Emilio M Osman; Cemic (Buenos Aires): Mariano G Setten, Pablo Lovazzano; Hospital Universitrario Austral (Pilar): Javier Alvarez, Veronica Villar; Hospital Por + Salud (Pami) Dr. Cesar Milstein (Buenos Aires): Norberto C Pozo, Nicolas Grubissich; Sanatorio Anchorena (Buenos Aires): Gustavo A Plotnikow, Daniela N Vasquez; Sanatorio De La Trinidad Mitre (Buenos Aires): Santiago Ilutovich, Norberto Tiribelli; Hospital Luis Lagomaggiore (Mendoza): Ariel Chena, Carlos A Pellegrini; H.I.G.A San Martín (La Plata): María G Saenz, Elisa Estenssoro; Hospital Misericordia (Cordoba): Matias Brizuela, Hernan Gianinetto; Sanatorio Juncal (Temperley): Pablo E Gomez, Valeria I Cerrato; Hospital D. F. Santojanni (Buenos Aires): Marco G Bezzi, Silvina A Borello; Hospital Alejandro Posadas (Buenos Aires): Flavia A Loiacono, Adriana M Fernandez;

**AUSTRALIA**: St. Vincents Hospital, Sydney (Darlinghurst): Serena Knowles, Claire Reynolds; St George Public Hospital (Kogarah): Deborah M Inskip, Jennene J Miller; Westmead Hospital (Westmead): Jing Kong, Christina Whitehead; Flinders Medical Centre (Bedford Park, South Australia): Shailesh Bihari; John Hunter Hospital (Newcastle): Aylin Seven, Amanda Krstevski; Canberra Hospital (Garran): Helen J Rodgers, Rebecca T Millar; Calvary Mater Newcastle (Waratah): Toni E Mckenna, Irene M Bailey; Cabrini Hospital (Melbourne): Gabrielle C Hanlon; Liverpool Hospital (Liverpool): Anders Aneman, Joan M Lynch; Coffs Harbour Health Campus (Coffs Harbour): Raman Azad, John Neal; Sir Charles Gairdner Hospital (Nedlands): Paul W Woods, Brigit L Roberts; Concord Hospital (Concord): Mark R Kol, Helen S Wong;

**AUSTRIA:** General Hospital Of Vienna/Medical University Of Vienna (Vienna): Katharina C Riss, Thomas Staudinger;

**BELGIUM**: Cliniques universitaires St Luc, UCL (Brussels): Xavier Wittebole, Caroline Berghe; CHU Dinant-Godinne (Yvoir): Pierre A Bulpa, Alain M Dive; AZ Sint Augustinus Veurne (Veurne): Rik Verstraete, Herve Lebbinck; Ghent University Hospital (Ghent): Pieter Depuydt, Joris Vermassen;; University Hospitals Leuven (Leuven): Philippe, Meersseman, Helga Ceunen;

**BRAZIL:** Hospital Renascentista (Pouso Alegre): Jonas I Rosa, Daniel O Beraldo; Vitoria Apart Hospital (Serra): Claudio Piras, Adenilton M Rampinelli; Hospital Das Clinicas (São Paulo): Antonio P Nassar Jr; Hospital Geral Do Grajaù (São Paulo): Sergio Mataloun, Marcelo Moock; Evangelical Hospital (Cachoeiro De Itapemirim / Espírito Santo): Marlus M Thompson, Claudio H Gonçalves-,; Hospital Moinhos De Vento (Porto Alegre): Ana Carolina P Ant ônio, Aline Ascoli; Hospital Alvorada Taguatinga (Taguatinga): Rodrigo S Biondi, Danielle C Fontenele; Complexo Hospitalar Mngabeira Tarcisio Burity (Joao Pessoa): Danielle Nobrega, Vanessa M Sales;

**BRUNEI DARUSSALAM:** Raja Isteri Pengiran Anak Saleha (Ripas) Hospital (Bandar Seri Begawan): Dr Suresh .Shindhe, Dr Dk Maizatul Aiman B Pg Hj Ismail;

**CANADA:** Medical-Surgical ICU of St Michael’s Hospital (Toronto): John Laffey, Francois Beloncle; St. Josephs Health Centre (Toronto): Kyle G Davies, Rob Cirone; Sunnybrook Health Sciences Center (Toronto): Venika Manoharan, Mehvish Ismail; Toronto Western Hospital (Toronto): Ewan C Goligher, Mandeep Jassal; Medical Surgical ICU of the Toronto General Hospital (Toronto): Niall D. Ferguson, Erin Nishikawa, Areej Javeed; Cardiovascular ICU of St Michael’s Hospital (Toronto): Gerard Curley, Nuttapol Rittayamai ; Cardiovascular ICU of the Toronto General Hospital (Toronto): Matteo Parotto, Mount Sinai Hospital (Toronto): Sangeeta Mehta, Jenny Knoll ; Trauma-Neuro ICU of St Michael’s Hospital (Toronto): Antoine Pronovost, Sergio Canestrini

**CHILE:** Hospital Clínico Pontificia Universidad Católica De Chile (Santiago): Alejandro R Bruhn, Patricio H Garcia; Hospital Militar De Santiago (Santiago): Felipe A Aliaga, Pamela A Farías; Clinica Davila (Santiago): Jacob S Yumha; Hospital Guillermo Grant Benavente (Concepcion): Claudia A Ortiz, Javier E Salas; Clinica Las Lilas (Santiago): Alejandro A Saez, Luis D Vega; Hospital Naval Almirante Nef (Viña Del Mar): Eduardo F Labarca, Felipe T Martinez; Hospital Luis Tisné Brousse (Penanolen): Nicolás G Carreño, Pilar Lora;

**CHINA:** The Second Affiliated Hospital Of Harbin Medical University (Harbin): Haitao Liu; Nanjing Zhong-Da Hospital, Southeast University (Nanjing): Haibo Qiu, Ling Liu; The First Affiliated Hospital Of Anhui Medical University (Hefei): Rui / Tang, Xiaoming Luo; Peking University People’s Hospital (Beijing): Youzhong An, Huiying Zhao; Fourth Affiliated Hospital Of Harbin Medical University (Harbin): Yan - Gao, Zhe - Zhai; Nanjing Jiangbei Peoples Hospital Affiliated To Medical School Of Southeast University (Nanjing): Zheng L Ye, Wei Wang; The First Affiliated Hospital Of Dalian Medical Unvercity (Dalian): Wenwen Li, Qingdong Li; Subei Peoples Hospital Of Jiangsu Province (Yanghzou): Ruiqiang Zheng ; Jinling Hospital (Nanjing): Wenkui Yu, Juanhong Shen; Urumqi General Hospital (Urumqi): Xinyu Li; Intensive Care Unit, First Affiliated Hospital Of Wanna Medical College, Yijishan Hospital, (Wuhu): Tao Yu, Weihua Lu; Sichuan Provincial Peoples Hospital (Chengdu): Ya Q Wu, Xiao B Huang; Hainan Province Peoples Hospital (Haikou): Zhenyang He; Peoples Hospital Of Jiangxi Province (Nanchang): Yuanhua Lu; Qilu Hospital Of Shandong University (Jinan): Hui Han, Fan Zhang; Zhejiang Provincial Peoples Hospital (Hangzhou): Renhua Sun ; The First Affiliated Hospital Of Bengbu Medical College (Bengbu, Anhui): Hua X Wang, Shu H Qin; Nanjing Municipal Government Hospital (Nanjing): Bao H Zhu, Jun Zhao; The First Hospital Of Lanzhou University (Lanzhou): Jian / Liu, Bin / Li; The First Affiliated Hospital Of Chongqing University Of Medical Science (Chongqing): Jing L Liu, Fa C Zhou; Xuzhou Central Hospital, Jiangsu Province, China (Xuzhou): Qiong J Li, Xing Y Zhang; The First Peoples Hospital Of Foshan (Foshan): Zhou Li-Xin, Qiang Xin-Hua; The First Affiliated Hospital Of Guangxi Medical University (Nanning): Liangyan Jiang; Renji Hospital ,Shanghai Jiao Tong University School Of Medicine (Shanghai): Yuan N Gao, Xian Y Zhao; First Hospital Of Shanxi Medical University (Taiyuan): Yuan Y Li, Xiao L Li; Shandong Provincial Hospital (Jinan): Chunting Wang, Qingchun Yao ; Fujian Provincial Hospital (Fuzhou): Rongguo Yu, Kai Chen; Henan Provincial People’s Hospital (Zhengzhou): Huanzhang Shao, Bingyu Qin ; The Second Affiliated Hospital Of Kunming Medical University (Kunming City): Qing Q Huang, Wei H Zhu; Xiangya Hospital, Central South University (Changsha): Ai Y Hang, Ma X Hua; The First Affiliated Hospital Of Guangzhou Medical University (Guangzhou): Yimin Li, Yonghao Xu; Peoples Hospital of Hebei Province (Shijiazhuang): Yu D Di, Long L Ling; Guangdong General Hospital (Guangzhou): Tie H Qin, Shou H Wang; Beijing Tongren Hospital (Beijing): Junping Qin; Jiangsu Province Hospital (Nanjing): Yi Han, Suming Zhou;COLOMBIA: Fundación Valle Del Lili (Cali): Monica P Vargas;

**COSTA RICA**: Hospital San Juan De Dios (): Juan I Silesky Jimenez, Manuel A González Rojas; Hospital San Juan De Dios (San José): Jaime E Solis-Quesada, Christian M Ramirez-Alfaro;

**CZECH REPUBLIC**: University Hospital Of Ostrava (Ostrava): Jan Máca, Peter Sklienka;

**DENMARK:** Aarhus Universitetshospital (Aarhus N): Jakob Gjedsted, Aage Christiansen; Rigshopitalet: Jonas Nielsen;

**ECUADOR:** Hospital Militar (Quito): Boris G Villamagua, Miguel Llano;

**FRANCE:** Clinique du Millénaire (Montpellier): Philippe Burtin, Gautier Buzancais; Centre Hospitalier (Roanne): Pascal Beuret, Nicolas Pelletier; CHU d’Angers (Angers): Satar Mortaza, Alain Mercat; Hôpital Marc Jacquet (Melun): Jonathan Chelly, Sébastien Jochmans; CHU de Caen (Caen): Nicolas Terzi, Cédric Daubin; Henri Mondor Hospital (Créteil): Guillaume Carteaux, Nicolas de Prost; Cochin Hospital (Paris): Jean-Daniel Chiche, Fabrice Daviaud ; CHU Tenon (Paris): Tài Pham, Muriel Fartoukh; CH Mulhouse-Emile Muller (Mulhouse): Guillaume Barberet, Jerome Biehler; Archet 1 University Hospital (Nice): Jean Dellamonica, Denis Doyen; Hopital Sainte Musse (Toulon): Jean-Michel Arnal, Anais Briquet; Hopital Nord - Réanimation des Détresses Respiratoires et Infections Sévères (Marseille): Fanny Klasen, Laurent Papazian; HEGP (Paris): Arnaud Follin; Louis Mourier Hospital (Colombes): Damien Roux, Jonathan Messika; Centre Hospitalier de Dax (Dax): Evangelos Kalaitzis ; Réanimation Médicale, GH Pitié-Salpêtrière (Paris): Laurence Dangers, Alain Combes; Ap-Hp Ambroise Paré (Boulogne-Billancourt): Siu-Ming Au; University Hospital Rouen (Rouen): Gaetan Béduneau, Dorothée Carpentier; CHU Amiens (Amiens - Salouel): Elie H Zogheib, Herve Dupont; Centre Hospitalier Intercommunal Robert Ballanger (Aulnay Sous Bois): Sylvie Ricome, Francesco L Santoli; Centre Hospitalier René Dubos (Pontoise): Sebastien L Besset; CHI Portes de l’Oise (Beaumont Sur Oise): Philippe Michel, Bruno Gelée; Archet 2 University Hospital (Nice): Pierre-Eric Danin, Bernard Goubaux; Centre Hospitalier Pierre Oudot (Bourgoin Jallieu): Philippe J Crova, Nga T Phan; CH Dunkerque (Dunkerque): Frantz Berkelmans; Centre Hospitalier de Belfort Montbéliard (Belfort): Julio C Badie, Romain Tapponnier; Centre Hospitalier Emile Muller (Mulhouse): Josette Gally, Samy Khebbeb; Hôpital de Hautepierre-Hôpitaux Universitaires de Strasbourg (Strasbourg): Jean-Etienne Herbrecht, Francis Schneider; Centre Hospitalier de Dieppe (Dieppe): Pierre-Louis M Declercq, Jean-Philippe Rigaud; Bicêtre (Le Kremin-Bicetre): Jacques Duranteau, Anatole Harrois; CHU Gabriel Montpied (Clermont-Ferrand): Russell Chabanne, Julien Marin; CHU Estaing (Clermont-Ferrand): Jean-Michel Constantin, Sandrine Thibault; CHI Eure-Seine Evreux (Evreux): Mohammed Ghazi, Messabi Boukhazna; Centre Hospitalier de Châlons en Champagne (Châlons en Champagne): Salem Ould Zein; CH Beauvais (Beauvais): Jack R Richecoeur, Daniele M Combaux; Centre Hospitalier Le Mans (Le Mans): Fabien Grelon, Charlene Le Moal; Hôpital Fleyriat (Bourg en Bresse): Elise P Sauvadet, Adrien Robine; Hôpital Saint Louis (Paris): Virginie Lemiale, Danielle Reuter; Pneumologie et Réanimation Médicale, Hôpital Pitié-Salpêtrière (Paris): Martin Dres, Alexandre Demoule; Centre Hospitalier Gonesse (Gonesse): Dany Goldgran-Toledano; Hôpital Croix Rousse (Lyon): Loredana Baboi, Claude Guérin;

**GERMANY:** St. Nikolaus-Stiftshospital (Andernach): Ralph Lohner; Fachkrankenhaus Coswig Gmbh (Coswig):Jens Kraßler, Susanne Schäfer; University Hospital Frankfurt (Frankfurt am Main): Kai D Zacharowski, Patrick Meybohm; Department of Anaesthesia & Intensive Care Medicine, University Hospital of Leipzig (Leipzig): Andreas W Reske, Philipp Simon; Asklepios Klinik Langen (Langen): Hans-Bernd F Hopf, Michael Schuetz; Städtisches Krankenhaus Heinsberg (Heinsberg): Thomas Baltus;

**GREECE**: Hippokrateion General Hospital Of Athens (Athens): Metaxia N Papanikolaou, Theonymfi G Papavasilopoulou; Gh Ahepa (Thessaloniki): Giannis A Zacharas, Vasilis Ourailogloy; Hippokration General Hospital of Thessaloniki (Thessaloniki): Eleni K Mouloudi, Eleni V Massa; Hospital General of Kavala (Kavala): Eva O Nagy, Electra E Stamou; Papageorgiou General Hospital (Thessaloniki): Ellada V Kiourtzieva, Marina A Oikonomou;

**GUATEMALA**: Hospital General De Enfermedades, Instituto Guatemalteco De Seguridad Social (Ciudad De Guatemala): Luis E Avila; Centro Médico Militar (Guatemala): Cesar A Cortez, Johanna E Citalán;

**INDIA**: Deenanath Mangeshkar Hospital And Research Center (Pune): Sameer A Jog, Safal D Sable; Care Institute Of Medical Sciences (CIMS) Hospital (Ahmedabad): Bhagyesh Shah ; Sanjay Gandhi Postgraduate Institute Of Medical Sciences (SGPGIMS) (Lucknow): Mohan Gurjar, Arvind K Baronia; Rajasthan Hospital (Ahmedabad): Mohammedfaruk Memon ; National Institute Of Mental Health And Neuro Sciences (NIMHANS) (Bangalore): Radhakrishnan Muthuchellappan, Venkatapura J Ramesh; Anaesthesiology Unit of the Kasturba Medical College & Dept of Respiratory Therapy, SHOAS, Manipal University (Manipal): Anitha Shenoy, Ramesh Unnikrishnan; Sanjeevan Hospital (Pune): Subhal B Dixit, Rachana V Rhayakar; Apollo Hospitals (Chennai): Nagarajan Ramakrishnan ,Vallish K Bhardwaj; Medicine Unit of the Kasturba Medical College & Dept of Respiratory Therapy, SHOAS, Manipal University (Manipal): Heera L Mahto, Sudha V Sagar; G Kuppuswamy Naidu Memorial Hospital (Coimbatore): Vijayanand Palaniswamy, Deeban Ganesan;

**IRAN:** NRITLD/Masih Daneshvari (Tehran): Seyed Mohammadreza Hashemian, Hamidreza Jamaati ; Milad Hospital (Tehran): Farshad Heidari

**IRELAND**: St Vincent’s University Hospital (Dublin): Edel A Meaney, Alistair Nichol; Mercy University Hospital (Cork): Karl M Knapman, Donall O’Croinin ; Cork University Hospital (Cork): Eimhin S Dunne, Dorothy M Breen; Galway University Hospital (Galway): Kevin P Clarkson, Rola F Jaafar; Beaumont Hospital (Dublin): Rory Dwyer, Fahd Amir; Mater Misericordiae University Hospital (Dublin): Olaitan O Ajetunmobi, Aogan C O’Muircheartaigh; Tallaght Hospital (Dublin): Colin S Black, Nuala Treanor; Saint James’s Hospital (Dublin): Daniel V Collins, Wahid Altaf;

**ITALY**: Santa Maria delle Croci Hospital (Ravenna): Gianluca Zani, Maurizio Fusari; Arcispedale Sant’Anna Ferrara. (Ferrara): Savino Spadaro, Carlo A Volta; Ospedale Profili (Fabriano) (An): Romano Graziani, Barbara Brunettini; Umberto I Nocera Inferiore (Nocera Inferiore Salerno): Salvatore Palmese; Azienda Ospedaliera San Paolo – Polo Universitario- Università degli Studi di Milano (Milan): Paolo Formenti, Michele Umbrello; Sant’Anna (San Fermo Della Battaglia (Co)): Andrea Lombardo; Spedali Civili Brescia (Brescia): Elisabetta Pecci, Marco Botteri; Fondazione Irccs Ca Granda, Ospedale Maggiore Policlinico (Milan): Monica Savioli, Alessandro Protti; University Campus Bio-Medico of Rome (Rome): Alessia Mattei, Lorenzo Schiavoni; Azienda Ospedaliera "Mellino Mellini" (Chiari (Bs)): Andrea Tinnirello, Manuel Todeschini; Policlinico P. Giaccone, University of Palermo (Palermo): Antonino Giarratano, Andrea Cortegiani; Niguarda Cà Granda Hospital (Milan): Sara Sher, Anna Rossi; A.Gemelli University Hospital (Rome): Massimo M Antonelli, Luca M Montini; Ospedale "Sandro Pertini" (Rome): Paolo Casalena, Sergio Scafetti; ISMeTT IRCCS UPMC

(Palermo): Giovanna Panarello, Giovanna Occhipinti; Ospedale San Gerardo (Monza): Nicolò Patroniti, Matteo Pozzi; Santa Maria Della Scaletta (Imola): Roberto R Biscione, Michela M Poli; Humanitas Research Hospital (Rozzano): Ferdinando Raimondi, Daniela Albiero; Ospedale Desio - Ao Desio-Vimercate (Desio): Giulia Crapelli, Eduardo Beck; Pinetagrande Private Hospital (Castelvolturno): Vincenzo Pota, Vincenzo Schiavone; Irccs San Martino Ist (Genova): Alexandre Molin, Fabio Tarantino; Ospedale San Raffaele (Milano): Giacomo Monti, Elena Frati; Ospedali Riuniti Di Foggia (Foggia): Lucia Mirabella, Gilda Cinnella; Azienda Ospedaliera Luigi Sacco - Polo Universitario (Milano): Tommaso Fossali, Riccardo Colombo; A.O.U. Città della Salute e della Scienza di Torino (Turin): Pierpaolo Terragni Ilaria Pattarino; Università degli Studi di Pavia-Fondazione IRCCS Policlinico San Matteo (Pavia): Francesco Mojoli, Antonio Braschi; Ao Ospedale Civile Legnano (Legnano): Erika E Borotto; Arnas Ospedale Civico Di Cristina Benfratelli (Palermo): Andrea N Cracchiolo, Daniela M Palma; Azienda Ospedaliera Della Provincia Di Lecco - Ospedale "A. Manzoni" (Lecco): Francesco Raponi, Giuseppe Foti; A.O. Provincia Di Lecco - Ospedale Alessandro Manzoni (Lecco): Ettore R Vascotto, Andrea Coppadoro; Cliniche Universitarie Sassari (Sassari): Luca Brazzi, Leda Floris; IRCCS Policlinico San Matteo (Pavia): Giorgio A Iotti, Aaron Venti;

**JAPAN**: Yokohama City University Hospital (Yokohama): Osamu Yamaguchi, Shunsuke Takagi; Toyooka Hospital (Toyooka City,Hyogo Prefecture): Hiroki N Maeyama; Chiba University Hospital (Chiba City): Eizo Watanabe, Yoshihiro Yamaji; Okayma University Hospital (Okayama): Kazuyoshi Shimizu, Kyoko Shiozaki; Japanese Foundation for Cancer Research, Cancer Institute Hospital, Department Of Emergency Medicine And Critical Care (Tokyo): Satoru Futami; Ibaraki Prefectural Central Hospital (Kasama): Sekine Ryosuke; Tohoku University Hospital (Sendai-Shi): Koji Saito, Yoshinobu Kameyama; Tokyo Medical University Hachioji Medical Center (Hachioji, Tokyo): Keiko Ueno; Tokushima University Hospital (Tokushima): Masayo . Izawa, Nao Okuda; Maebashi Red Cross Hospital (Gunma Maebashi): Hiroyuki Suzuki, Tomofumi Harasawa; Urasoe General Hospital (Urasoe): Michitaka Nasu, Tadaaki Takada; Ohta General Hospital Foundation Ohta Nishinouchi Hospital (Fukushima): Fumihito Ito; Jichi Medical University Hospital (Shimotsuke): Shin - Nunomiya, Kansuke - Koyama; Mito Kyodo General Hospital, Tsukuba University Hospital Mito Medical Center (Mito): Toshikazu Abe; Sendai City Hospital (Sendai): Kohkichi Andoh, Kohei Kusumoto; Ja Hiroshima General Hospital (Hatsukaichi City, Hiroshima): Akira Hirata, Akihiro Takaba; Yokohama Rosai Hospital (Yokohama): Hiroyasu Kimura; Nagasaki University Hospital (Nagasaki): Shuhei Matsumoto, Ushio Higashijima; Niigata University Medical & Dental Hospital (Niigata): Hiroyuki Honda, Nobumasa Aoki; Mie University Hospital (Tsu, Mie): Hiroshi Imai; Yamaguchi University Hospital (Ube, Yamaguchi): Yasuaki Ogino, Ichiko Mizuguchi; Saiseikai Kumamoto Hospital (Kumamoto City): Kazuya Ichikado; Shinshu University School Of Medecine (Matsumoto City): Kenichi Nitta, Katsunori Mochizuki; Kuki General Hospital (Kuki): Tomoaki Hashida; Kyoto Medical Center (Kyoto): Hiroyuki Tanaka ; Fujita Health University (Toyoake): Tomoyuki Nakamura, Daisuke Niimi; Rakwakai Marutamachi Hospital (Kyoto): Takeshi Ueda; Osaka University Hospital (Suita City, Osaka Prefecture): Yozo Kashiwa, Akinori Uchiyama;

**LATVIA**: Paul Stradins Clinical University Hospital (Riga): Olegs Sabelnikovs , Peteris Oss;

**LEBANON**: Kortbawi Hospital (Jounieh): Youssef Haddad;

**MALAYSIA**: Hospital Kapit (Kapit): Kong Y Liew;

**MEXICO**: Instituto Nacional De Cancerología, México (Mexico City): Silvio A Ñamendys-Silva, Yves D Jarquin-Badiola; Hospital De Especialidades "Antonio Fraga Mouret" Centro Medico Nacional La Raza IMSS (Mexico City): Luis A Sanchez-Hurtado, Saira S Gomez-

Flores; Hospital Regional 1° De Octubre (Mexico City): Maria C Marin, Asisclo J Villagomez; Hospital General Dr Manuel Gea Gonzalez (Mexico City): Jordana S Lemus, Jonathan M Fierro; Hospital General De Zona No. 1 Instituto Mexicano Del Seguro Social Tepic Nayarit (Tepic): Mavy Ramirez Cervantes, Francisco Javier Flores Mejia; Centro Medico Dalinde (Mexico D.F.): Dulce Dector, Dulce M Dector; Opd Hospital Civil De Guadalajara Hospital Juan I Menchaca (Guadalajara): Daniel R Gonzalez, Claudia R Estrella; Hospital Regional De Ciudad Madero Pemex (Ciudad Madero): Jorge R Sanchez-Medina, Alvaro Ramirez-Gutierrez; Centro Médico ABC (Mexico D.F.): Fernando G George, Janet S Aguirre; Hospital Juarez De Mexico (Mexico City): Juan A Buensuseso, Manuel Poblano;

**MOROCCO**: Mohammed V University, University Teaching Ibn Sina Hospital (Rabat): Tarek Dendane, Amine Ali Zeggwagh; Hopital Militaire D’Instruction Mohammed V (Rabat): Hicham Balkhi; Errazi (Marrakech): Mina Elkhayari, Nacer Samkaoui; University Teaching Hospital Ibn Rushd (Casablanca): Hanane Ezzouine, Abdellatif Benslama; Hôpital des Spécialités de Rabat (HSR) (Rabat): Mourad Amor, Wajdi Maazouzi;

**NETHERLANDS**: Tjongerschans (Heerenveen): Nedim Cimic, Oliver Beck; Cwz (Nijmegen): Monique M Bruns, Jeroen A Schouten; Rijnstate Hospital (Arnhem): Myra - Rinia, Monique Raaijmakers; Radboud Umc (Nijmegen): Leo M Heunks, Hellen M Van Wezel; Maastricht University Medical Centre (Maastricht): Serge J Heines, Ulrich Strauch; Catharinaziekenhuis (Eindhoven): Marc P Buise; Academic Medical Center (Amsterdam): Fabienne D Simonis, Marcus J Schultz;

**NEW ZEALAND**: Tauranga Hospital (Tauranga): Jennifer C Goodson, Troy S Browne; Wellington Hospital (Wellington): Leanlove Navarra, Anna Hunt; Dunedin Hospital (Dunedin): Robyn A Hutchison, Mathew B Bailey; Auckland City Hospital (Auckland): Lynette Newby, Colin Mcarthur; Whangarei Base Hospital (Whangarei): Michael Kalkoff, Alex Mcleod; North Shore Hospital (Auckland): Jonathan Casement, Danielle J Hacking;

**NORWAY**: Ålesund Hospital (Ålesund): Finn H Andersen, Merete S Dolva; Oslo University Hospital - Rikshospitalet Medical Centre (Oslo): Jon H Laake, Andreas Barratt-Due; Stavanger University Hospital (Stavanger): Kim Andre L Noremark, Eldar Søreide; Haukeland University Hospital (Bergen): Brit Å Sjøbø, Anne B Guttormsen;

**PERU**: Hospital Nacional Edgardo Rebagliati Martins (Lima): Hector H Leon Yoshido; Clínica Ricardo Palma (Lima): Ronald Zumaran Aguilar, Fredy A Montes Oscanoa;

**PHILIPPINES**: The Medical City (Pasig): Alain U Alisasis, Joanne B Robles; Chong Hua Hospital (Cebu): Rossini Abbie B Pasanting-Lim, Beatriz C Tan;

**POLAND**: Warsaw University Hospital (Warsaw): Pawel Andruszkiewicz, Karina Jakubowska;

**PORTUGAL**: Centro Hospitalar Da Cova Da Beira (Covilhã): Cristina M Coxo; Hospital Santa Maria, Chln (Lisboa): António M Alvarez, Bruno S Oliveira; Centro Hospitalar Trás-Os-Montes E Alto Douro - Hospital De S.Pedro -Vila Real (Vila Real): Gustavo M Montanha, Nelson C Barros; Hospital Beatriz Ângelo (Loures): Carlos S Pereira, António M Messias; Hospital De Santa Maria (Lisboa): Jorge M Monteiro; Centro Hospitalar Médio Tejo - Hospital De Abrantes (Abrantes): Ana M Araujo, Nuno T Catorze; Instituto Português De Oncologia De Lisboa (Lisboa): Susan M Marum, Maria J Bouw; Hospital Garcia De Orta (Almada): Rui M Gomes, Vania A Brito; Centro Hospitalar Do Algarve (Faro): Silvia Castro, Joana M Estilita; Hpp Hospital De Cascais (Alcabideche): Filipa M Barros; Hospital Prof. Doutor Fernando Fonseca Epe (Amadora): Isabel M Serra, Aurelia M Martinho;

**ROMANIA**: Fundeni Clinical Institute (Bucharest): Dana R Tomescu, Alexandra Marcu; Emergency Clinical County Hospital Timisoara (Timisoara): Ovidiu H Bedreag, Marius

Papurica; Elias University Emergency Hospital (Bucharest): Dan E Corneci, Silvius Ioan Negoita;

**RUSSIAN FEDERATION**: University Hospital (Kemerovo): Evgeny Grigoriev ;Krasnoyarsk Regional Hospital, Krasnoyarsk State Medical University (Krasnoyarsk): Alexey I Gritsan, Andrey A Gazenkampf;

**SAUDI ARABIA:** GICU of PSMMC (Riyadh): *Ghaleb Almekhlafi, Mohamad M Albarrak*; SICU of PSMMC (Riyadh): Ghanem M Mustafa;; King Faisal Hospital And Research Center (Riyadh): Khalid A Maghrabi, Nawal Salahuddin; King Fahad Hospital (Baha): Tharwat M Aisa; King Abdulaziz Medical City (Riyadh): Ahmed S Al Jabbary, Edgardo Tabhan; King Abdulaziz Medical City (Riyadh): Yaseen M Arabi; King Abdulaziz Medical City (Riyadh): Yaseen M Arabi, Olivia A Trinidad; King Abdulaziz Medical City (Riyadh): Hasan M Al Dorzi, Edgardo E Tabhan;

**SOUTH AFRICA:** Charlotte Maxeke Johannesburg Academic Hospital (Johannesburg): Stefan Bolon, Oliver Smith;

**SPAIN**: Hospital Sant Pau (Barcelona): Jordi Mancebo, Hernan Aguirre-Bermeo; Hospital Universitari Bellvitge (L Hospitalet De Llobregat (Barcelona)): Juan C Lopez-Delgado, Francisco Esteve; Hospital Son Llatzer (Palma De Mallorca): Gemma Rialp, Catalina Forteza; Sabadell Hospital, CIBER Enfermedades Respiratorias (Sabadell): Candelaria De Haro, Antonio Artigas; Hospital Universitario Central De Asturias (Oviedo): Guillermo M Albaiceta, Sara De Cima-Iglesias; Complejo Hospitalario Universitario A Coruña (A Coruña): Leticia Seoane-Quiroga, Alexandra Ceniceros-Barros; Hospital Universitario Miguel Servet (Zaragoza): Antonio L Ruiz-Aguilar, Luis M Claraco-Vega; Morales Meseguer University Hospital (Murcia): Juan Alfonso Soler, Maria del Carmen Lorente; Hospital Universitario del Henares (Coslada): Cecilia Hermosa, Federico Gordo; Complejo Asistencial De Palencia. Hospital Rio Carrión (Palencia): Miryam - Prieto-González, Juan B López-Messa; Fundación Jiménez Díaz (Madrid): Manuel P Perez, Cesar P Perez; Hospital Clínico Universitario Lozano Blesa (Zaragoza): Raquel Montoiro Allue; Hospital Verge de la Cinta (Tortosa): Ferran Roche-Campo, Marcos Ibañez-Santacruz; Hospital Universitario 12 De Octubre (Madrid): Susana - Temprano; Hospital Universitario Príncipe De Asturias (Alcalá De Henares, Madrid): Maria C Pintado, Raul De Pablo; Hospital Universitari Germans Trias I Pujol (Badalona): Pilar Ricart Aroa Gómez; Hospital Universitario Arnau De Vilanova De Lleida (Lleida): Silvia Rodriguez Ruiz, Silvia Iglesias Moles; Cst Terrassa (Barcelona): Mª Teresa Jurado, Alfons Arizmendi; Hospital Universitari Mútua Terrassa (Terrassa): Enrique A Piacentini; Hospital Universitario De Móstoles (Mostoles): Nieves Franco, Teresa Honrubia; Complejo Asistencial De Salamanca (Salamanca): Meisy Perez Cheng, Elena Perez Losada; Hospital General Universitario De Ciudad Real (Ciudad Real): Javier - Blanco, Luis J Yuste; Torrecardenas (Almeria): Cecilia Carbayo-Gorriz, Francisca G Cazorla-Barranquero; Hospital Universitario Donostia (San Sebastian): Javier G Alonso, Rosa S Alda; Hospital Universitario De Torrejón (Madrid): Ángela Algaba, Gonzalo Navarro; Hospital Universitario De La Princesa (Madrid): Enrique Cereijo, Esther Diaz-Rodriguez; Hospital Universitario Lucus Augusti (Lugo): Diego Pastor Marcos, Laura Alvarez Montero; Hospital Universitario Santa Lucia (Cartagena): Luis Herrera Para, Roberto Jimenez Sanchez; Hospital Universitario Severo Ochoa, Leganes (Madrid): Miguel Angel Blasco Navalpotro, Ricardo Diaz Abad; University Hospital Of Ntra. Sra. De Candelaria (Santa Cruz De Tenerife): Raquel Montiel Gonz á lez, D á cil Parrilla Toribio; Hospital Universitario Marques De Valdecilla (Santander): Alejandro G Castro, Maria Jose D Artiga; Hospital Infanta Cristina (Parla, Madrid): Oscar Penuelas ; Hospital General De Catalunya (Sant Cugat Del Valles): Tomas P Roser, Moreno F Olga; San Pedro De Alcántara (Cáceres): Elena Gallego Curto, Rocío Manzano Sánchez; Sant Joan De Reus (Reus): Vallverdu P Imma, Garcia

M Elisabet; Hospital Joan XXIII (Tarragona): Laura Claverias, Monica Magret; Hospital Universitario De Getafe (Madrid): Ana M Pellicer, Lucia L Rodriguez; Hospital Universitario Río Hortega (Valladolid): Jesús Sánchez-Ballesteros, Ángela González-Salamanca; Hospital Arquitecto Marcide (Ferrol,La Coruña): Antonio G Jimenez, Francisco P Huerta; Hospital General Universitario Gregorio Marañón (Madrid): Juan Carlos J Sotillo Diaz, Esther Bermejo Lopez;Hospital General De Segovia (Segovia): David D Llinares Moya, Alec A Tallet Alfonso; Hospital General Universitario Reina Sofia (Murcia): Palazon Sanchez Eugenio Luis, Palazon Sanchez Cesar; Complejo Hospitalario Universitario De Albacete (Albacete): Sánchez I Rafael, Corcoles G Virgilio; Hospital Infanta Elena (Valdemoro): Noelia N Recio;

**SWEDEN**: Sahlgrenska University Hospital (Gothenburg): Richard O Adamsson, Christian C Rylander; Karolinska University Hospital (Stockholm): Bernhard Holzgraefe, Lars M Broman; Akademiska Sjukhuset Uppsala (Uppsala): Joanna Wessbergh, Linnea Persson; Vrinnevisjukhuset (Norrköping): Fredrik Schiöler, Hans Kedelv; Linkoping University Hospital (Linköping): Anna Oscarsson Tibblin, Henrik Appelberg; Skellefteå Lasarett (Skellefteå): Lars Hedlund, Johan Helleberg; Karolinska University Hospital Solna (Stockholm): Karin E Eriksson, Rita Glietsch; Umeå University Hospital (Umeå): Niklas Larsson, Ingela Nygren; Danderyd Hospital (Stockholm): Silvia L Nunes, Anna-Karin Morin; Lund University Hospital (Lund): Thomas Kander, Anne Adolfsson;

**SWITZERLAND**: Chuv (Centre Hospitalier Universitaire Vaudois) (Lausanne): Lise Piquilloud; Hôpital neuchâtelois - La Chaux-De-Fonds (La Chaux-De-Fonds): Hervé O. Zender, Corinne Leemann-Refondini;

**TUNISIA**: Hopital Taher Sfar Mahdia (Mahdia): Souheil Elatrous; University Hospital Farhat Hached Sousse (Sousse): Slaheddine Bouchoucha, Imed Chouchene; CHU F.Bourguiba (Monastir): Islem Ouanes; Mongi Slim University Hospital, La Marsa (La Marsa): Asma Ben Souissi, Salma Kamoun;

**TURKEY**: Cerrahpasa Medical Faculty Emergency Intensive Care Unit (Istanbul): Oktay Demirkiran; Cerrahpasa Medical Faculty Sadi Sun Intensive Care Unit (Istanbul) : Mustafa Aker, Emre Erbabacan; Uludag University Medical Faculty (Bursa): Ilkay Ceylan, Nermin Kelebek Girgin; Ankara University Faculty of Medicine, Reanimation 3nd level ICU (Ankara): Menekse Ozcelik, Necmettin Ünal; Ankara University Faculty of Medicine, 2nd level ICU-postoperative ICU (Ankara): Basak Ceyda Meco; Istanbul Kartal Egitim Ve Arastirma Hastanesi (Istanbul): Onat O Akyol, Suleyman S Derman;

**UNITED KINGDOM**: Papworth Hospital (Cambridge): Barry Kennedy, Ken Parhar; Royal Glamorgan Hospital (Llantrisant): Latha Srinivasa; Royal Victoria Hospital-Belfast (Belfast): Lia McNamee, Danny McAuley; Jack Steinberg ICU of the King’s College (London): Phil Hopkins, Clare Mellis; Frank Stansil ICU of the King’s College Hospital (London): Vivek Kakar; ;Liver ICU of the King’s College (London): Dan Hadfield; Christine Brown ICU of the King’s College (London): Andre Vercueil; West Suffolk Hospital (Bury St Edmunds): Kaushik Bhowmick, Sally K Humphreys; Craigavon Area Hospital (Portadown): Andrew Ferguson, Raymond Mckee; Barts Health NHS Trust, Whipps Cross Hospital (Leytonstone): Ashok S Raj, Danielle A Fawkes; Kettering General Hospital, Foundation NHS Trust (Northamptonshire): Philip Watt, Linda Twohey; Barnet General Hospital (Barnet): Rajeev R JhaMatthew Thomas, Alex Morton, Varsha Kadaba; Rotherham General Hospital (Rotherham): Mark J Smith, Anil P Hormis; City Hospital, (Birmingham): Santhana G Kannan, Miriam Namih; Poole Hospital NHS Foundation Trust (Poole): Henrik Reschreiter, Julie Camsooksai; Weston General Hospital (Weston-Super-Mare): Alek Kumar, Szabolcs Rugonfalvi; Antrim Area Hospital (Antrim): Christopher Nutt, Orla Oneill; Aintree University Hospital (Liverpool): Colette Seasman, Ged Dempsey; Northern General Hospital (Sheffield): Christopher J Scott, Helen E Ellis; John Radcliffe Hospital (Oxford): Stuart Mckechnie, Paula J Hutton; St Georges Hospital (London): Nora N Di Tomasso, Michela N Vitale; Hillingdon Hospital (Uxbridge): Ruth 0 Griffin, Michael N Dean; The Royal Bournemouth & Christchurch NHS Foundation Trust (Bournemouth, Dorset): Julius H Cranshaw, Emma L Willett; Guys And St Thomas NHS Foundation Trust (London): Nicholas Ioannou, Gstt Severe Respiratory Failure Service ; Whittington Hospital (London): Sarah Gillis; Wexham Park Hospital (Slough): Peter Csabi; Western General Hospital (Edinburgh): Rosaleen Macfadyen, Heidi Dawson; Royal Preston Hospital (Preston): Pieter D Preez, Alexandra J Williams; Brighton And Sussex University Hospitals NHS Trust (Brighton): Owen Boyd, Laura Ortiz-Ruiz De Gordoa; East And North Herts NHS Trust (Stevenage): Jon Bramall, Sophie Symmonds; Barnsley Hospital (Barnsley): Simon K Chau, Tim Wenham; Prince Charles Hospital (Merthyr Tydfil): Tamas Szakmany, Piroska Toth-Tarsoly; University Hospital Of South Manchester NHS Foundation Trust (Manchester): Katie H Mccalman, Peter Alexander; Harrogate District Hospital (Harrogate): Lorraine Stephenson, Thomas Collyer; East And North Herts NHS Trust (Welwyn Garden City): Rhiannon Chapman, Raphael Cooper; Western Infirmary (Glasgow): Russell M Allan, Malcolm Sim; Dumfries And Galloway Royal Infirmary (Dumfries): David W Wrathall, Donald A Irvine; Charing Cross Hospital (London): Kim S Zantua, John C Adams; Worcestershire Royal Hospital (Worcester): Andrew J Burtenshaw, Gareth P Sellors; Royal Liverpool University Hospital (Liverpool): Ingeborg D Welters, Karen E Williams; Royal Alexandra Hospital (Glasgow): Robert J Hessell, Matthew G Oldroyd; Morriston Hospital (Swansea): Ceri E Battle, Suresh Pillai; Frimley Park Hospital (Frimley): Istvan - Kajtor, Mageswaran - Sivashanmugavel; Altnagelvin Hospital (Derry): Sinead C Okane, Adrian Donnelly; Buckinghamshire Healthcare NHS Trust (High Wycombe, Buckinghamshire): Aniko D Frigyik, Jon P Careless; Milton Keynes Hospital (Milton Keynes): Martin M May, Richard Stewart; Ulster Hospital (Belfast): T John Trinder, Samantha J Hagan; University Hospital of Wales (Cardiff): Matt P Wise, Jade M Cole; Freeman Hospital (Newcastle Upon Tyne): Caroline C MacFie, Anna T Dowling;

**URUGUAY**: Hospital Español (Montevideo): Javier Hurtado, Nicolás Nin; Cudam (Montevideo): Javier Hurtado; Sanatorio Mautone (Maldonado): Edgardo Nuñez ; Sanatorio Americano (Montevideo): Gustavo Pittini, Ruben Rodriguez; Hospital De Clínicas (Montevideo): María C Imperio, Cristina Santos; Circulo Católico Obreros Uruguay- Sanatorio JPII (Montevido: Ana G. França, Alejandro EBEID; CASMU (Montevideo): Alberto Deicas, Carolina Serra

**USA**: Saint Louis University Hospital (St.Louis): Aditya Uppalapati, Ghassan Kamel; Beth Israel Deaconess Medical Center (Boston): Valerie M Banner-Goodspeed, Jeremy R Beitler; Memorial Medical Center (Springfield): Satyanarayana Reddy Mukkera, Shreedhar Kulkarni; Massachusetts General Hospital (Boston): Jarone Lee, Tomaz Mesar; University Of Cincinnati Medical Center (Cincinnati): John O Shinn Iii, Dina - Gomaa; Massachusetts General Hospital (Boston): Christopher Tainter, Jarone Lee; Massachusetts General Hospital (Boston): Tomaz Mesar, Jarone Lee; R Adams Cowley Shock Trauma Center (Baltimore): Dale J Yeatts, Jessica Warren; Intermountain Medical Center (Murray, Utah): Michael J Lanspa, Russel R Miller; Intermountain Medical Center (Murray, Utah): Colin K Grissom, Samuel M Brown; Mayo Clinic (Rochester): Philippe R Bauer; North Shore Medical Center (Salem): Ryan J Gosselin, Barrett T Kitch; Albany Medical Center (Albany): Jason E Cohen, Scott H Beegle; John H Stoger Hospital Of Cook County (Chicago, Il): Renaud M Gueret, Aiman Tulaimat; Albany Medical Center (Albany): Shazia Choudry ; University of Alabama at Birmingham (UAb) (Birmingham, AL): William Stigler, Hitesh Batra ; Duke University Hospital (Durham): Nidhi G Huff; Iowa Methodist Medical Center (Des Moines, Iowa): Keith D Lamb, Trevor W Oetting; Surgical & Neurosciences Intensive Care Unit of the University Of Iowa Hospitals And Clinics (Iowa City, Iowa): Nicholas M Mohr, Claine Judy; Medical Center of Louisiana at New Orleans (New Orleans, Louisiana): Shigeki Saito, Fayez M Kheir; Tulane University (New Orleans): Fayez Kheir; Critical Care Unit of the University Of Iowa Hospitals And Clinics (Iowa City, Iowa): Adam B Schlichting, Angela Delsing; University Of California, San Diego Medical Center (San Diego, Ca): Daniel R Crouch, Mary Elmasri; Uc San Diego Thornton Hospital (La Jolla): Daniel R Crouch, Dina Ismail; University Hospital (Cincinnati): Kyle R Dreyer, Thomas C Blakeman; University Hospital (Cincinnati): Kyle R Dreyer, Dina Gomaa; Tower 3B Medical ICU of Brigham and Women’s Hospital (Boston): Rebecca M Baron, Carolina Quintana Grijalba; Tower 8C Burn/Trauma ICU of Brigham and Women’s Hospital (Boston): Peter C Hou; Tower 8D Surgical ICU of Brigham and Women’s Hospital (Boston): Raghu Seethala; Tower 9C Neurosurgical ICU of Brigham and Women’s Hospital (Boston): Imo Aisiku; Tower 9D Neurological ICU of Brigham and Women’s Hospital (Boston): Galen Henderson; Tower 11C Thoracic ICU of Brigham and Women’s Hospital (Boston): Gyorgy Frendl; Shapiro 6W Cardiac Surgery ICU of Brigham and Women’s Hospital (Boston): Sen-Kuang Hou; Shapiro 9E Coronary Care Unit of Brigham and Women’s Hospital (Boston): Robert L Owens, Ashley Schomer;

**SERBIA**: Clinical Center of Serbia (Belgrade): Vesna Bumbasirevic, Bojan Jovanovic; ; Military Medical Academy (Belgrade): Maja Surbatovic, Milic Veljovic;

**e-Appendix 2:** Additional data collected for patients having no ARDS risk factors

**FORM 4C: IF PATIENT HAD NO RISK FACTOR FOR ARDS**

**Was a broncho-alveolar lavage (BAL) fluid analysis performed?** 🞏 Yes 🞏 No

If yes, please provide

- - Day BAL performed: _ _ / _ _ / _ _ _ _
  - Cytological analysis:

Macroscopic aspect: 🞏 normal 🞏 bloody or pink 🞏 lactescent

Number of cells: _ _ _ _ _ _ / mL

Macrophages: _ _ % lymphocytes: _ _ % neutrophils: _ _ %

mast cells: _ _ % eosinophils: _ _ % siderophages: _ _ % other cells: _ _ %

- - Microbiological analyses performed (check all that apply):

🞏 Bacterial culture

🞏 *Pneumocystis jiroveci* stain or PCR

🞏 Fungal analysis

🞏 Viral PCRs

Positive result: _ _ _ _ _

**Were immunological tests performed?** 🞏 Yes 🞏 No

If yes, please check if the result is positive:

🞏 antinuclear antibodies

🞏 Antisynthetase antibodies

🞏 Anti-CCP antibody

🞏 ANCA

🞏 Rheumatoid factor

🞏 Other: _ _ _ _

**Was the patient taking pneumotoxic medications* before the development of ARDS?** 🞏 Yes 🞏 No

If yes, provide name of the drugs (check all that apply)

🞏 Amiodarone

🞏 Chemotherapy agents: _ _ _ _ _ _ _ _

🞏 Methotrexate

🞏 Hydrochlorothiazide

🞏 Tyrosine kinase inhibitors

🞏 Other: _ _ _ _ _ _ _ _ _

* see [www.pneumotox.com](http://www.pneumotox.com) for more information

**Was a final etiology for ARDS obtained?** 🞏 Yes 🞏 No

If yes, specify: _ _ _ _ _ _ _ _ _ _ _ _ _ _ _ _ _ _ _ _ _ _ _ _ _ _ _ _ _ _ _ _ _ _ _ _ _ _ _ _

**Was a chest CT-scan performed?** 🞏 Yes 🞏 No

If yes, day chest CT-scan performed: _ _ / _ _ / _ _ _ _

If yes, provide CT-scan patterns present (check all that apply):

🞏 *Honeycombing*

🞏 *Ground class attenuation*

🞏 *Traction bronchiectasis*

🞏 *Interlobular septal thickening*

🞏 *Air space consolidation* *including atelectasis*

🞏 *Other* Specify: _ _ _ _ _ _ _ _ _ _ _ _ _ _ _ _ _ _ _ _ _ _ _ _ _ _ _ _ _ _ _ _ _ _

**Was a lung biopsy performed?** 🞏 Yes 🞏 No

If yes, day lung biopsy performed: _ _ / _ _ / _ _ _ _

If yes, provide histopathological pattern:

🞏 *Diffuse alveolar damage*

🞏 *Diffuse alveolar hemorrhage*

🞏 *Eosinophilic infiltration*

🞏 *Bronchiolitis obliterans organizing pneumonia*

🞏 *Lung fibrosis*

🞏 *Other* Specify: _ _ _ _ _ _ _ _ _ _ _ _ _ _ _ _ _ _ _ _ _ _ _ _ _ _ _ _ _ _ _ _ _ _
